# T Cell Specific BOB.1/OBF.1 Expression Promotes Germinal Center Response and T Helper Cell Differentiation

**DOI:** 10.3389/fimmu.2022.889564

**Published:** 2022-05-04

**Authors:** Annika C. Betzler, Jasmin Ezić, Tsima Abou Kors, Thomas K. Hoffmann, Thomas Wirth, Cornelia Brunner

**Affiliations:** ^1^ Department of Oto-Rhino-Laryngology, Ulm University Medical Center, Ulm, Germany; ^2^ Department of Physiological Chemistry, Ulm University, Ulm, Germany

**Keywords:** BOB.1/OBF.1, germinal center, T cell differentiation, follicular T helper cells, humoral immunity, transcriptional co-activator, Pou2af1

## Abstract

The transcriptional co-activator BOB.1/OBF.1 is expressed in both B and T cells. The main characteristic of conventional BOB.1/OBF.1 deficient mice is the complete absence of germinal centers (GCs). This defect was mainly attributed to the defective B cell compartment. However, it is unknown whether and how BOB.1/OBF.1 expression in T cells contributes to the GC reaction. To finally clarify this question, we studied the *in vivo* function of BOB.1/OBF.1 in CD4^+^ T and follicular T helper (TFH) cell subpopulations by conditional mutagenesis, in the presence of immunocompetent B lymphocytes. BOB.1/OBF.1 deletion in CD4^+^ T as well as TFH cells resulted in impaired GC formation demonstrating that the impaired GC reaction described for conventional BOB.1/OBF.1-deficient mice cannot exclusively be traced back to the B cell compartment. Furthermore, we show a requirement of BOB.1/OBF.1 for T helper (TH) cell subsets, particularly for TFH cell differentiation.

## Introduction

BOB.1/OBF.1 (also known as OCA-B), encoded by the *Pou2af1* (POU domain class 2-associating factor 1) gene, is a lymphocyte-restricted transcriptional co-activator involved in octamer-dependent transcription (1). The octamer motif (5’-ATGCAAAT-3’) is found in all immunoglobulin (Ig) heavy and light chain gene promoters as well as enhancer elements (2, 3). Octamer-dependent transcription is achieved by transcription factors (TFs) belonging to the Oct-family including Oct-1 and Oct-2. The Oct-1 protein is ubiquitously expressed, whereas the Oct-2 protein is specific for cells of hematopoietic origin (4). The binding selectivity to the octamer motif and the transcriptional activity of these Oct transcriptions factors can be enhanced by the additional recruitment of BOB.1/OBF.1. The transcriptional regulator BOB.1/OBF.1 itself has a low affinity to DNA but is instead recruited to the octamer motif by its direct interaction with Oct TFs (5–8). Thus, BOB.1/OBF.1 activates octamer-dependent transcription by its synergistic interaction with Oct proteins, thereby regulating the expression of genes essential for lymphocyte physiology. BOB.1/OBF.1 has long been considered a B cell specific factor and is mainly studied in B cells. Thereby it has been shown that BOB.1/OBF.1 is constitutively expressed in B lymphocytes, where its highest expression level was observed in germinal center (GC) B cells (9, 10). BOB.1/OBF.1 deficiency leads to B cell developmental defects in the bone marrow as well as to later defects observed in the periphery (11–14). BOB.1/OBF.1-deficient mice feature severely reduced splenic B cells and impaired follicular B cell maturation (11, 14, 15). BOB.1/OBF.1 is additionally required for marginal zone B cell development and function (13). The hallmark of BOB.1/OBF.1-deficient animals is the complete absence of GCs and consequently severely reduced levels of secondary Igs and the failure to generate post-GC B cells (14, 16), caused by the combined binding of BOB.1/OBF.1 together with Oct TFs to genes important for GC formation in mouse and human GC B cells controlling their transcriptional program (17).

On the other hand, the role and function of BOB.1/OBF.1 in T cells is poorly understood. In CD4^+^ T cells, BOB.1/OBF.1 expression can be induced upon treatment with PMA/Ionomycin or antigen receptor engagement (18–20). There is evidence that NFAT, together with NFκB TFs, controls the expression of BOB.1/OBF.1 thereby regulating octamer-dependent transcription in T cells (20). Previously, we observed a role of BOB.1/OBF.1 to control TH1/TH2 cytokine balance and its influence on *IL-2* and *INFγ* promoter activities (21). Moreover, reports from our and other groups revealed that the development and function of follicular T helper (TFH) cells involve BOB.1/OBF.1 (22, 23). Thus, a direct contribution of BOB.1/OBF.1 to the promoter activity of *Bcl6*, the master regulator of TFH cell lineage, was shown (22). In addition, a requirement of BOB.1/OBF.1 together with Oct-1 for the generation and function of CD4^+^ memory T cells was suggested (24). Most recently, a role for BOB.1/OBF.1 in TH17 differentiation and in the regulation of IL17A production was described (25, 26).

Over the last years, a role for BOB.1/OBF.1 also in the human immune system has become more and more evident. A patient with a mutation in the *Pou2af1* gene leading to the absence of BOB.1/OBF.1 protein was described with B cell developmental defects, agammaglobulinemia and a reduction in circulating TFH cells (27). Beyond that, dysregulated expression of BOB.1/OBF.1 in both B and T cells was described to contribute to the pathogenesis of several autoimmune diseases including Rheumatoid arthritis, Type 1 diabetes, multiple sclerosis and systemic lupus erythematosus (28–30). Thus, BOB.1/OBF.1 might be a promising pharmacological target for certain immune-related diseases.

Therefore, a better understanding of BOB.1/OBF.1 functions and its possible target genes is necessary. Especially, the question whether the expression of BOB.1/OBF.1 in T cells contributes to the GC reaction is still controversially discussed (9, 22, 31). To finally clarify whether and how BOB.1/OBF.1 expression in T cells contributes to the GC reaction, we now generated a mouse model enabling the regulation of BOB.1/OBF.1 expression specifically in defined T cell subpopulations that contribute to the GC reaction, in the presence of a functional B cell compartment.

Here we studied the *in vivo* function of BOB.1/OBF.1 in distinct T cell subpopulations by conditional mutagenesis. Using this mouse system, we analyzed the consequences of deleting BOB.1/OBF.1 precisely in CD4^+^ T cells as well as TFH cells. Contrary to previous assumptions, we provide evidence that the impaired GC reaction described for conventional BOB.1/OBF.1-deficient mice cannot exclusively be traced back to the B cell compartment, since BOB.1/OBF.1 deletion in CD4^+^ T as well as TFH cells also resulted in impaired GC formation. Furthermore, our data show a contribution of BOB.1/OBF.1 for T helper (TH) and especially TFH cell differentiation. RNA-seq analysis identified several genes among the top 30 differentially expressed genes (DEGs) in Pou2af1^fl/fl^ x CD4-Cre mice involved in T cell differentiation and function.

## Material and Methods

### Mice and Immunization

Mice were bred on the C57BL/6JRj background and maintained under specific pathogen-free conditions at the animal facility of Ulm University. The Pou2af1^fl/fl^ mice (C57BL/6JRj background) were crossed to CD4-Cre (32) or IL21-Cre mice (33). Animal experimentation was done in accordance with German legislation for animal protection (reference number: 35/9185.81-3/1325). Mice were maintained in a specific pathogen-free facility under standard housing conditions with continuous access to food and water. Mice used in the study were 8-14-weeks old and were maintained on 12 h light, 12 h dark light cycle (6:00-18:00). To test T-dependent antibody responses and Germinal Center reaction, mice were immunized with 10^8^ Sheep Red Blood Cells (SRBCs) (Cedarlane Laboratories, Canada) in PBS by i.p. injection. To analyze antibody production during a secondary immune response, mice were immunized on day 0 and again on day 14. Blood was collected on days 0, 14 and 21. To assess antigen-specific antibody levels, mice were immunized with 100 µg NP-KLH (Biosearch Technologies) emulsified in Imject Alum (Thermo Fisher Scientific) by i.p injection. Serum was analyzed for the presence of antigen-specific immunoglobulins after 28 days.

### Generation of Floxed *Pou2af1* Alleles

The strategy for the generation of a conditional Pou2af1 allele is described elsewhere (34).

### T Cell Purification and Immunoblot Analysis

To examine the expression of BOB.1/OBF.1 in CD4^+^ T cells in the respective mouse strains, T cells were enriched by a magnetic column. Appropriate kits were used to isolate CD4^+^ T cells (Milteny Biotec). TFH cells were sorted on a BD FACS Aria cell sorter (BD Biosciences) based on their expression of CD3, CD4, CXCR5 and PD-1. Due to limited number of sorted TFH cells, cells were pooled from two or three mice of respective genotypes. Isolated or sorted cells were lysed in RIPA buffer and immunoblotting was performed following standard procedures using the following antibodies: anti-BOB.1/OBF.1 (self-made) and anti-β-actin (Sigma-Aldrich, Missouri, USA). Horseradish-peroxidase conjugated rabbit anti-mouse antibody was used as a secondary antibody. Membranes were developed with a chemiluminescence detection system (Bio-Rad, California, USA).

### Flow-Cytometric Analysis

The cell staining procedure was described earlier (34). Data were acquired on a Gallios cytometer (Beckman Coulter, California, USA) and analyzed with Kaluza Analysis software, version 2.1 (Beckman Coulter, California, USA). Used antibodies are summarized in **Table S1**. Intracellular Foxp3 staining was performed using the Treg detection kit (Milteny Biotec).

### Immunohistochemistry

Paraffin-embedded sections of spleens from immunized mice were stained with biotinylated Peanut Agglutinin (PNA) (Vector Laboratories, California, USA). This was followed by detection with horseradish-peroxidase conjugated streptavidin and AEC chromogen. Subsequently, slides were counter stained with hematoxylin.

### Enzyme-Linked Immunosorbent Assay (ELISA)

Immunoglobulin ELISA was performed as previously described (34). For determination of NP-specific antibody levels in sera from 28 d NP-KLH immunized mice ELISA plates were coated overnight at 4°C with 10 µg/ml NP(9)- and NP(27)-BSA. The next day, plates were blocked with 5% BSA in PBS overnight at 4°C. Subsequently, plates were washed, and serum samples were serially diluted in duplicate and incubated overnight at 4°C. Plates were then washed and incubated with alkaline phosphatase-conjugated goat anti-mouse IgM, IgG1 or IgG3 (SouthernBiotech, USA) diluted 1:1000 for 1h at 37°C. 4-nitrophenil phosphate-disodium salt (Serva, Germany) was used as substrate. Plates were read in a plate reader at 450 nm. Values were reported as relative absorbance.

### Cell Isolation, RNA Extraction and Sequencing

CD4^+^ T cells were isolated from LNs of CD4-Cre (n=4) and Pou2af1^fl/fl^ x CD4-Cre (n=9) mice after immunization with SRBCs by magnetic separation using CD4^+^ T cell isolation kit (Milteny Biotec). Purity of isolated CD4^+^ T cells was ≥ 96% for all samples and is shown in **Figure S14**. Extraction and purification of RNA were performed using RNeasy Mini Kit (Qiagen). RNA was eluted in RNase-free water and stored at -80°C. RNA quantity was measured using the Infinite 200 Pro (Tecan).

Quality was checked using an Agilent Tape Station and all samples displayed RIN values above 9. After quantity measurement by Qubit Fluorometer (Invitrogen) 200ng of RNA were used to generate sequencing libraries using the Illumina RNA-Seq Kit V2. Sequencing libraries were subsequently quality checked on D1000 screentapes (Agilent) and 10 samples each were sequenced using a NextSeq550 (Illumina) and 2 NextSeq 500/550 High Output Kits v2.5 (75 Cycles) at the Genomics Core Facility (Ulm University).

### Read Mapping and Quantification

Hiqh-quality reads were mapped to the mouse genome (GRChm38) using STAR (2.0.9). Followed by the removal of multimapping reads, converted to gene specific read counts for annotated genes using featureCounts (2.0.0).

### Differential Expression Analysis

Data analysis was performed in R (4.0.2) using the IDE Rstudio (1.3.1056). Differential expression analysis was performed using deseq2 (1.29.16), and the shrinkage estimator used was “apeglm” (35). For computation of distance matricies, euclidian distance was used. Generally, ggplot2 (3.3.2) was used for data visualization. The 99^th^ percentile of the log2foldchanges was calculated (**Figure S15**) and applied as a cutoff alongside an FDR cutoff of 0.05. This cutoff corresponds to an absolute foldchange of 1.1327. Clustering in the heatmaps was done using hierarchical clustering within the pheatmap (1.0.12) package. Gene ontology and visualization of the results was performed using clusterProfiler (3.18.0) and enrichplot (1.11.0.991). UMAP dimensionallity reduction has been performed using the package uwot (0.1.8), with the default parameters (except: n_neighbors = n/2). Waterfall plots were created using R (4.1.1) and the ggplot2 (3.3.5) package.

### Statistical Analysis

Results are expressed as arithmetic means ± standard deviation. Statistical analysis was performed with a two-tailed unpaired Student’s t-test or Mann-Whitney-U test as indicated, with GraphPad Prism V8 software. * p < 0.05, ** p < 0.01, *** p < 0.001, **** p < 0.0001.

## Results

### Conditional Deletion of Pou2af1 in CD4^+^ T Cells as Well as TFH Cells

**Figure 1:**
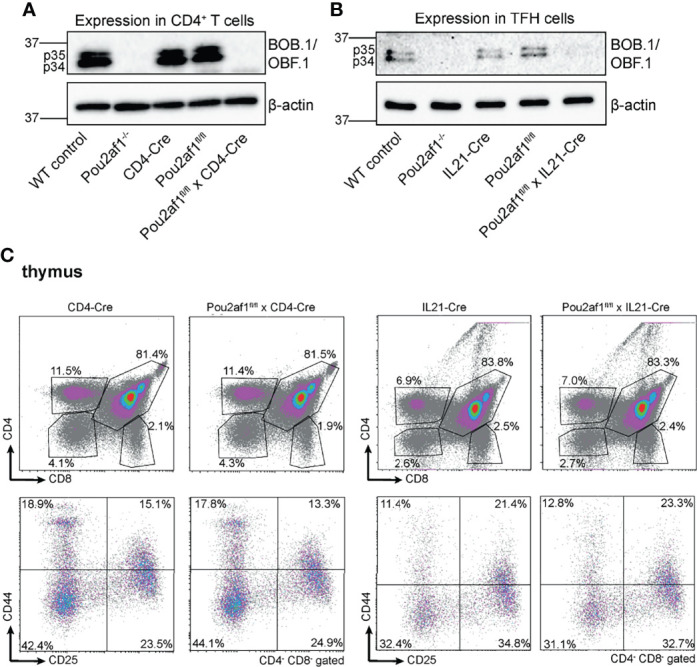
Conditional deletion of Pou2af1 in CD4^+^ T cells as well as TFH cells. **(A, B)** p34 and p35 BOB.1/OBF.1 isoform protein levels in CD4^+^ T **(A)** and TFH **(B)** cells were determined by immunoblotting, respectively. β-actin was used as loading control. **(C)** Representative flow cytometry plots of T cell development in the thymus of respective Cre-controls and conditional knockouts are shown. Gating strategy is shown in **Figure S7**.

To address the role of BOB.1/OBF.1 in T lymphocytes, we generated floxed *Pou2af1* alleles, as previously described (34). To assess the T lymphoid function of BOB.1/OBF.1, we used the CD4- (32) and IL21-Cre (33) mice. CD4-Cre was chosen to specifically delete BOB.1/OBF.1 in double positive and mature T cells. IL21-Cre mice were used to address the role of BOB.1/OBF.1 predominantly in TFH cells, key regulators of the GC response. IL21 is primarily produced by TFH cells, but a production from TH17 and natural killer T cells has also been reported (36, 37). Since IL21 is also required for TFH cell differentiation, it is already upregulated in developing pre-TFH cells (38). Thus, crossing Pou2af1^fl/fl^ mice to IL21-Cre mice results in BOB.1/OBF.1 deficiency already in pre-TFH cells and not specifically in fully developed TFH cells. The *Pou2af1* mRNA contains two translation initiation codons leading to the expression of two different isoforms – p40 and p34 (39). The transient p40-isoform serves as a precursor of a third p35 isoform, which is why predominantly the p34 and p35 isoform are detectable by immunoblot analysis. Conditional deletion of both p34 and p35 isoforms of BOB.1/OBF.1 in CD4^+^ T and TFH cells was confirmed by immunoblot analysis (**Figures 1A, B**). BOB.1/OBF.1 is completely absent in CD4^+^ T cells isolated from Pou2af1^fl/fl^ x CD4-Cre mice and CXCR5^+^ PD-1^+^ TFH cells isolated from Pou2af1^fl/fl^ x IL21-Cre mice. For all analyses, we compared the conditional knockouts to their respective Cre- and floxed-controls, ruling out any Cre- or floxed-dependent effects. Both Cre- and floxed-genes are functionally WT. For comparison, we included WT C57BL/6JRj and conventional Pou2af1 knockout mice generated by Nielsen et al., in which BOB.1/OBF.1 is deleted in all cell types permanently/stably (14). Both conditional knockouts are generally healthy and fertile. CD4-Cre or IL21-Cre expression itself have no effect on thymic T cell development (40) (**Figure 1C**).

### BOB.1/OBF.1 Deficiency in CD4^+^ T Cells Alters the CD4/CD8 T Cell Ratio in the Periphery

**Figure 2:**
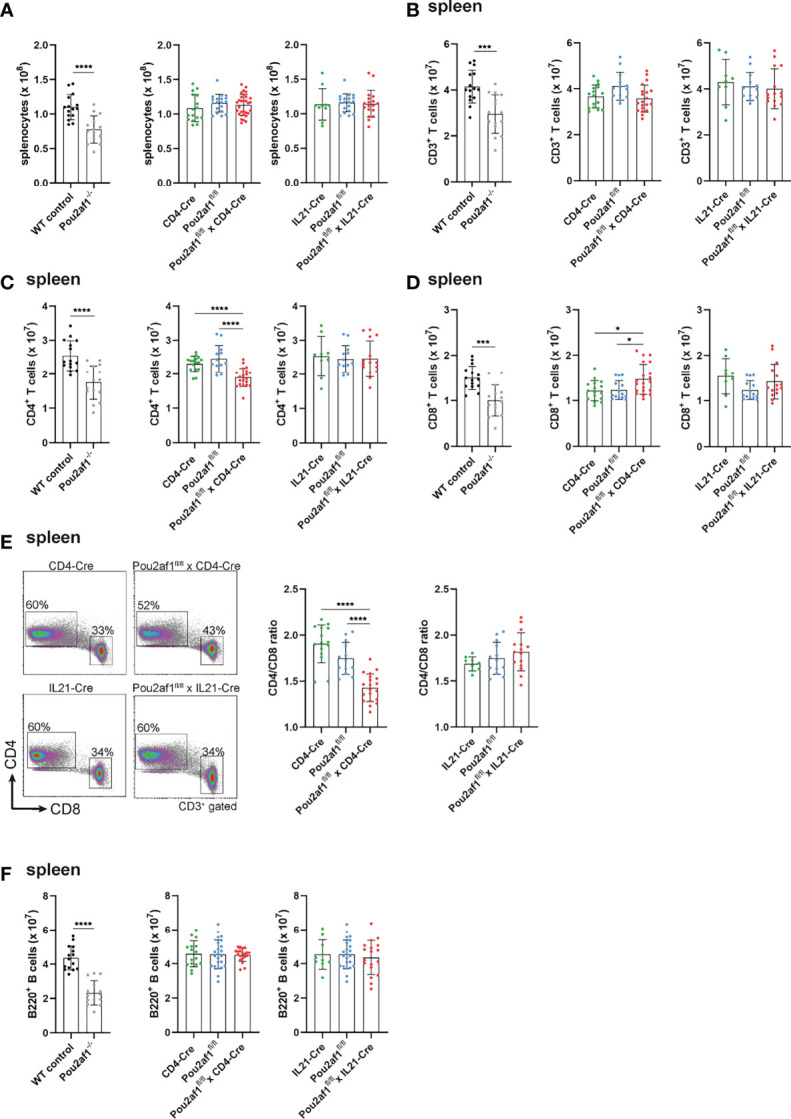
BOB.1/OBF.1 deficiency in CD4^+^ T cells alters the CD4/CD8 T cell ratio in the periphery. **(A)** Statistical representation of the number of splenocytes after lysis of erythrocytes. **(B–D)** Statistical representation of CD3^+^ **(B)**, CD4^+^ **(C)** and CD8^+^ **(D)** T cell numbers in the spleen assessed by flow cytometry. Each point represents data from a single mouse. Data in the graphs are shown as means ± SD (n = at least 13 mice per group). **(E)** Representative flow cytometry plots of CD4^+^ and CD8^+^ T cells and statstical analysis of the CD4/CD8 T cell ratio in the spleen. Gating strategy is shown in **Figure S8**. **(F)** Statistics of flow cytometric analyses showing B220^+^ B cell numbers in the spleen. Data are merged from at least three independent experiments. *p < 0.05, ***p < 0.001, and ****p < 0.0001. P-values were determined using a two-tailed Student’s t test or Mann-Whitney-U test.

First, we investigated the effect of conditional BOB.1/OBF.1 ablation on the T cell compartment in the periphery. Beforehand, numbers of splenocytes of both Pou2af1^fl/fl^ crossed either to CD4- or IL21-Cre mice were comparable to the WT situation (**Figure 2A**). Similarly, the overall number of CD3^+^ T cells was unaffected in both conditional knockouts in spleen and LNs (**Figure 2B**; **Figure S1A**). However, the number of CD4^+^ T cells was significantly reduced in Pou2af1^fl/fl^ x CD4-Cre mice in spleen and LN (**Figure 2C**; **Figure S1B**). The reduction of splenic CD4^+^ T cells observed in Pou2af1^fl/fl^ x CD4-Cre was comparable to conventional BOB.1/OBF.1-deficient animals. On the other hand, CD8^+^ T cell numbers were significantly increased in Pou2af1^fl/fl^ x CD4-Cre mice both in spleen and LN (**Figure 2D**; **Figure S1C**). Numbers of CD4^+^ and CD8^+^ T cells in Pou2af1^fl/fl^ x Il21-Cre mice were comparable to the WT situation (**Figures 2C, D**; **Figures S1B, C**). In accordance with cell numbers, Pou2af1^fl/fl^ x CD4-Cre mice featured markedly reduced percentages of CD4^+^ T cells, while at the same time increased percentages of CD8^+^ T cells resulting in a significantly altered CD4/CD8 T cell ratio in comparison to controls both in spleen and LN (**Figure 2E**; **Figure S1D**). The CD4/CD8 ratio in the spleen and LN of Pou2af1^fl/fl^ x Il21-Cre mice was unaffected (**Figure 2E**; **Figure S1D**). B cell numbers in both conditional knockouts were comparable to the WT situation in spleen and LN (**Figure 2F**; **Figure S1E**). These results suggest a role for BOB.1/OBF.1 in CD4^+^ T cell biology.

### CD4^+^ T- and TFH Cell-Specific BOB.1/OBF.1 Expression Is Required for Efficient GC Formation

**Figure 3:**
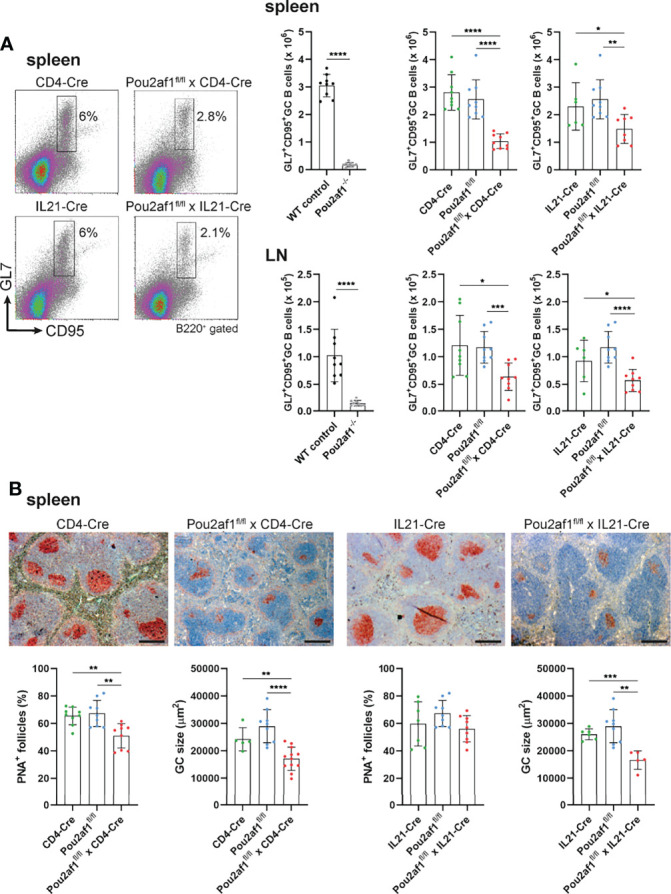
CD4^+^ T- and TFH cell-specific BOB.1/OBF.1 expression is required for efficient GC formation. **(A)** Representative flow cytometry plots and statistical analyses of GC B (GL7^+^ CD95^+^) cell numbers in spleen and LN are shown. Gating strategy is shown in **Figure S9**. **(B)** Representative immunohistochemical stainings of PNA^+^ GCs for each genotype and respective Cre-controls are shown. Scale bars indicate 200 µm. Statistical analyses showing PNA^+^ GCs and GC size stained by immunohistochemistry. Each point represents data from a single mouse. Data in the graphs are shown as means ± SD (n= 9 mice per group). Data are merged from two independent experiments. *p < 0.05, **p < 0.01, ***p < 0.001, and ****p < 0.0001. P-values were determined using a two-tailed Student’s t test or Mann-Whitney-U test.

To examine the contribution of T cell-specific BOB.1/OBF.1 expression for the GC reaction, we immunized Pou2af1^fl/fl^ mice crossed to either CD4- or IL21-Cre mice with Sheep Red Blood Cells (SRBCs). Flow-cytometric analyses of spleen and LN revealed significantly reduced levels of GL7^+^ CD95^+^ GC B cells after both CD4^+^ T and TFH cell-specific BOB.1/OBF.1 deletion (**Figure 3A**). GC B cell numbers were reduced by 60% in Pou2af1^fl/fl^ x CD4-Cre and by 35% in Pou2af1^fl/fl^ x IL21-Cre animals. Histological analysis of spleens confirmed the distinct reduction in the number of PNA^+^ GCs and revealed a significant decrease in GC size for Pou2af1^fl/fl^ x CD4-Cre mice (**Figure 3B**). In case of Pou2af1^fl/fl^ x IL21-Cre mice, no difference in the overall number of PNA^+^ stained GCs compared to controls was observed, but still the GC size was significantly reduced in the TFH cell-specific conditional knockout, which probably accounts for the diminished GC B cell numbers assessed by flow cytometry (**Figure 3B**). These findings demonstrate that the absence of GCs observed in conventional BOB.1/OBF.1-defcient mice cannot exclusively be traced back to the B cell compartment and defective B cell maturation. Our results provide evidence that specific deficiency of BOB.1/OBF.1 in T cells, while the B cell compartment stays immunocompetent, also impairs GC formation.

### BOB.1/OBF.1 Is Required for TFH Cell Development

**Figure 4:**
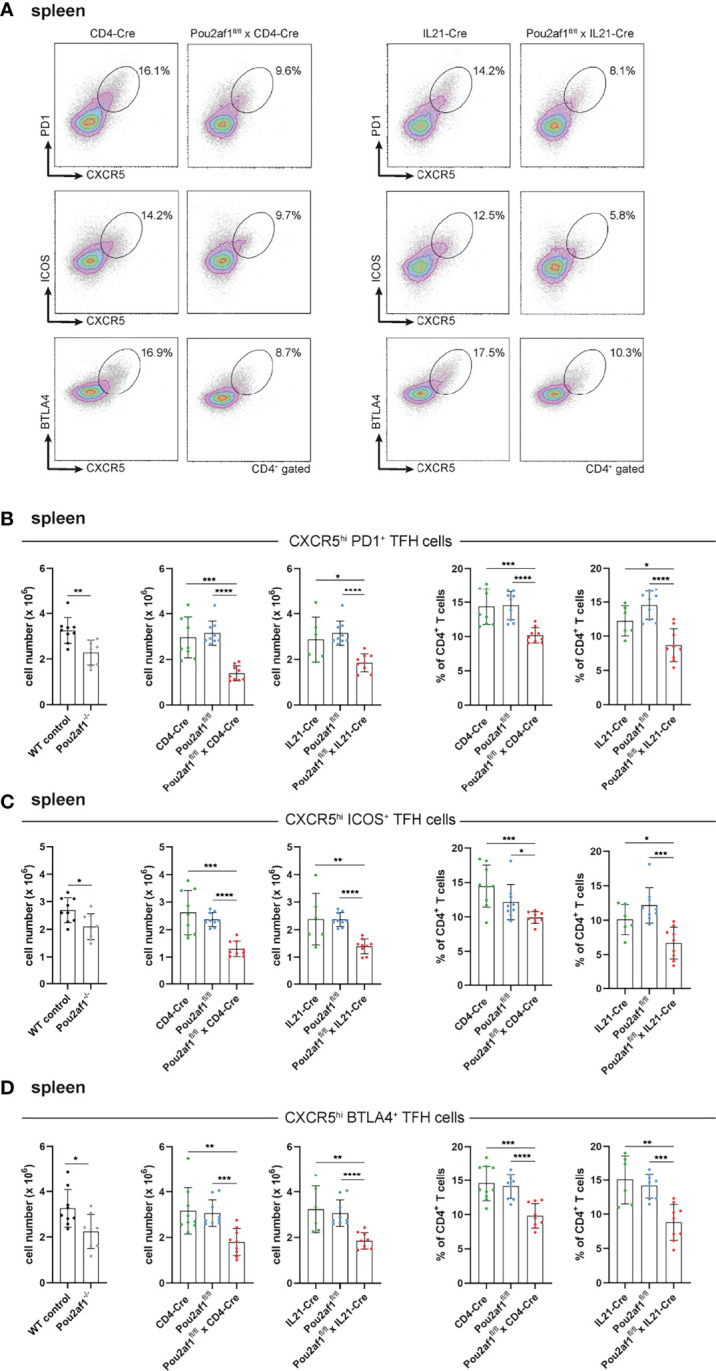
BOB.1/OBF.1 is required for TFH cell development. **(A–D)** Representative flow cytometry plots **(A)** and statistical analyses of CXCR5^high^ PD1^+^ **(B)**, CXCR5^high^ ICOS^+^ **(C)** and CXCR5^high^ BTLA4^+^ **(D)** TFH cell numbers (left panel) and TFH cell percentages (right panel) of CD4^+^ T cells in the spleen are shown. Gating strategy is shown in **Figure S10**. Each point represents data from a single mouse. Data in the graphs are shown as means ± SD (n= 9 mice per group). Data are merged from two independent experiments. *p < 0.05, **p < 0.01, ***p < 0.001, and ****p < 0.0001. P-values were determined using a two-tailed Student’s t test or Mann-Whitney-U test.

We and others have recently shown the impact of BOB.1/OBF.1 for TFH cell development and function (22, 23). TFH cells provide help for B cells regulating their proliferation and immunoglobulin class switching and are therefore relevant key player during GC reaction. TFH cells are characterized by the expression of the chemokine receptor CXCR5 together with at least one of the co-receptors PD1, ICOS or BTLA4 (22, 41). After immunization with SRBCs, both Pou2af1^fl/fl^ mice crossed to CD4- and IL21-Cre mice revealed significantly reduced levels of CXCR5^hi^ PD1^+^, CXCR5^hi^ ICOS^+^ and CXCR5^hi^ BTLA4^+^ TFH cells in the spleen and LN in comparison to respective controls (**Figures 4A–D**; **Figure S2**). In detail, in Pou2af1^fl/fl^ x CD4-Cre mice the frequency of CXCR5^hi^ PD1^+^ cells was reduced by 55%, of CXCR5^hi^ ICOS^+^ cells by 50% and of CXCR5^hi^ BTLA4^+^ cells by 30% in the spleen (**Figures 4B–D**). For Pou2af1^fl/fl^ x IL21-Cre mice a reduction by 40% of both CXCR5^hi^ PD1^+^ and CXCR5^hi^ ICOS^+^ cells was observed, while CXCR5^hi^ BTLA4^+^ cells were decreased by 35% (**Figures 4B–D**). Besides TFH cell numbers, also their percentage of CD4^+^ T cells was markedly reduced in both conditional knockouts in spleen and LNs (**Figure 4**; **Figure S2**). Together, these data reveal a requirement for BOB.1/OBF.1 for efficient TFH cell development during GC response. Reduced TFH cell frequencies in Pou2af1^fl/fl^ x IL21-Cre mice provide evidence for a TFH cell intrinsic defect in the absence of BOB.1/OBF.1 revealing that this reduction is not exclusively a consequence of defective CD4^+^ T cell differentiation towards TFH cells.

### Ablation of BOB.1/OBF.1 in CD4^+^ T Cells Results in Increased Treg Cell Levels and Impaired Antigen-Specific Immune Response

**Figure 5:**
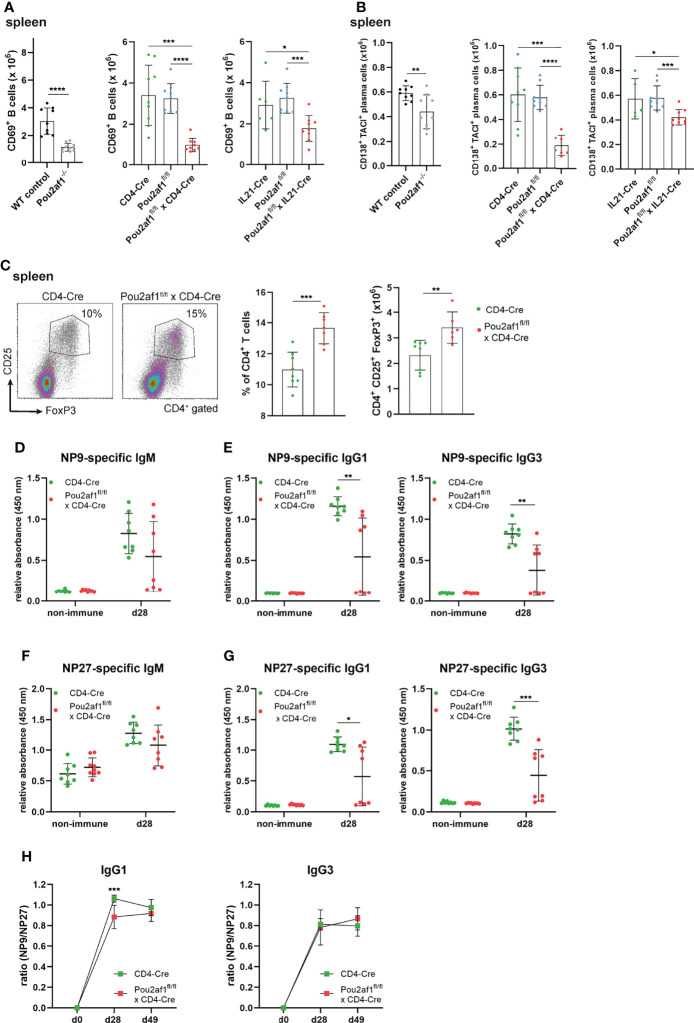
Ablation of BOB.1/OBF.1 in CD4^+^ T cells results in increased Treg cell levels and reduced antigen-specific immune response. **(A, B)** Statistics showing numbers of activated CD69^+^ B **(A)** and plasma **(B)** cells in the spleen after immunization. Gating strategy is shown in **Figures S11** and **S12** **(C)**. Representative flow cytometry plots and statistical analysis of Treg percentage and number in the spleen. Gating strategy is shown in **Figure S13** **(D–H)** ELISA of NP9-specific IgM **(D)**, NP9-specific IgG1 and IgG3 **(E)**, NP27-specific IgM **(F)** and NP27-specific IgG1 and IgG3 **(G)** in sera of mice before and after 28 days of NP-KLH immunization. Affinity maturation calculated by the ratio NP9/NP27 from day 0 to day 49 after immunization is shown **(H)**. Each point represents data from a single mouse. Data in the graphs are shown as means ± SD (n= at least 6 mice per group). Data are representative for two independent experiments. *p < 0.05, **p < 0.01, *** p< 0.001, and ****p < 0.0001. P-values were determined using a two-tailed Student’s t test or Mann-Whitney-U test.

Within GCs, B cells expressing high affinity antibodies develop and differentiate into antibody secreting plasma cells (42). We observed a significant reduction in the number of activated CD69^+^ B as well as antibody producing plasma cells in both conditional knockouts post immunization (**Figures 5A, B**). Since an efficient GC reaction results in the production of class-switched secondary Igs, we assessed IgM and class-switched IgG Ig levels in serum of the conditional knockout strains. For Pou2af1^fl/fl^ x CD4-Cre mice total Ig levels were assessed during a primary and secondary immune response and measured 0, 14 and 21 days after immunization by ELISA. Despite our findings of impaired GC formation, we could not detect a striking reduction in IgM or class-switched Igs in these mice (**Figure S3A**). Pou2af1^fl/fl^ x IL21-Cre mice revealed a reduction of IgM, IgG2b and IgG3 compared to the floxed-controls, but not when compared to Cre-controls (**Figure S3B**). Notably, we found a significantly increased frequency of regulatory T cells (Tregs) in Pou2af1^fl/fl^ x CD4-Cre mice compared to controls in spleen and LNs (**Figure 5C**; **Figure S3C**). Tregs are critical for the maintenance of immune homeostasis and regulate the initiation and input to the GC response (43). Since we observed increased levels of Tregs, we wondered about affinity maturation and subsequent production of antigen-specific Igs upon BOB.1/OBF.1 ablation in CD4^+^ T cells. Indeed, we could not detect significant differences in antigen-specific IgM levels, whereas levels of high-affinity anti-NP9 IgG1 and IgG3 were significantly reduced in Pou2af1^fl/fl^ x CD4-Cre mice 28 days after immunization with NP-KLH (**Figures 5D–F**). However, also levels of low affinity anti-NP27 antibodies of IgG1 and IgG3 isotype were significantly reduced in Pou2af1^fl/fl^ x CD4-Cre mice (**Figure 5G**). The ratio of NP9/NP27 antibody titers can be used as an index of affinity maturation. The ratio of high-affinity to low-affinity IgG1 was significantly reduced in the conditional knockout mice 28 days after immunization, even though this defect relativized on day 49 (**Figure 5H**). The ratio of NP9/NP27 IgG3 titers was not affected in Pou2af1^fl/fl^ x CD4-Cre mice (**Figure 5H**). In total, these data suggest a contribution of BOB.1/OBF.1 in T cells for the regulation of antigen-specific immune responses.

### Differential Gene Expression Between CD4^+^ T Cells of Pou2af1^fl/fl^ x CD4-Cre and Control Mice

**Figure 6:**
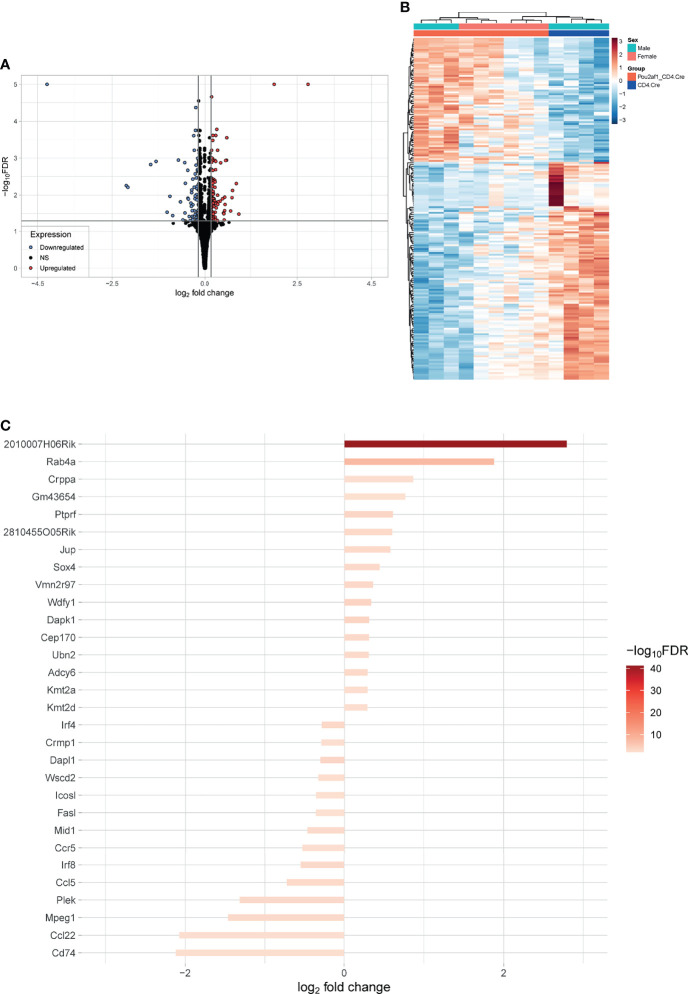
Differential gene expression between CD4^+^ T cells of Pou2af1^fl/fl^ x CD4-Cre and CD4-Cre control mice. CD4^+^ T cells from immunized Pou2af1^fl/fl^ x CD4-Cre and CD4-Cre controls were isolated from LNs and RNA-seq analysis was performed. **(A)** Volcano plot showing 158 DEGs at FDR 0.05 including 61 upregulated and 97 downregulated genes. The horizontal grey line indicates FDR cutoff of 0.05 and the vertical lines indicate the applied 99^th^ percentile cutoff of the log2foldchange. **(B)** Heatmap showing DEGs at FDR 0.01. **(C)** Waterfall plot showing the top 30 DEGs at FDR 0.01. Expression of *Pou2af1* was excluded from the top 30 DEGs.

To identify DEGs in CD4^+^ T cells in the absence of BOB.1/OBF.1 we isolated CD4^+^ T cells from LNs of Pou2af1^fl/fl^ x CD4-Cre and CD4-Cre controls after immunization with SRBCs and performed RNA-seq analysis. 158 genes were significantly differentially expressed (DEGs) in Pou2af1^fl/fl^ x CD4-Cre compared to controls at FDR 0.05 including 61 upregulated and 97 downregulated genes (**Figure 6A**; **Table S2**) Hierarchical clustering analysis suggested that certain groups of the differentially expressed genes might have similar functions or might be involved in the same pathways (**Figure 6B**; heatmap FDR 0.01).

**Figure 7:**
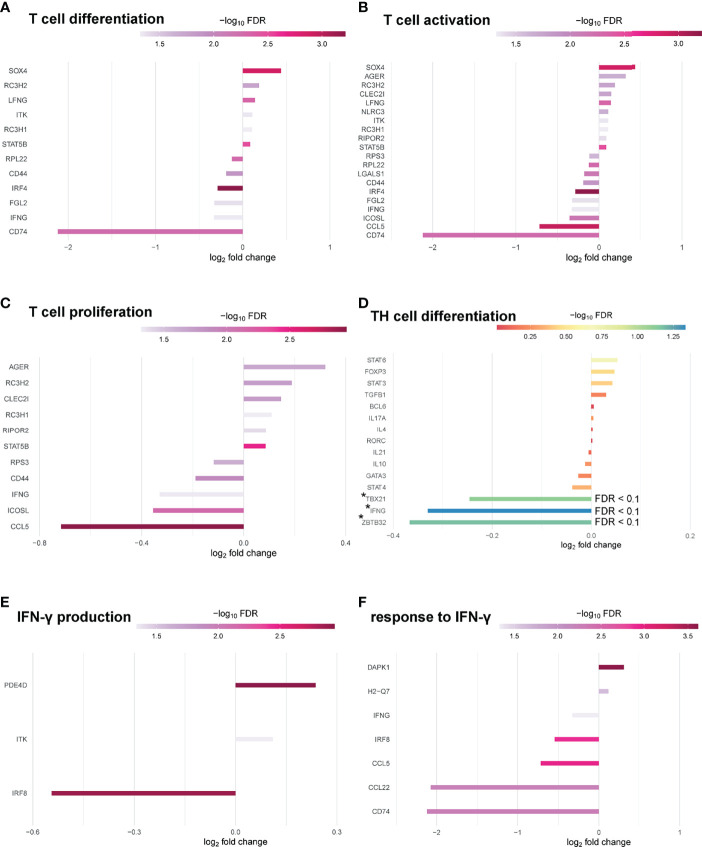
BOB.1/OBF.1 involved in TH cell differentiation. **(A–C)** Waterfall plots showing differentially expressed genes between Pou2af1^fl/fl^ x CD4-Cre and CD4-Cre control mice at FDR 0.05 related to T cell differentiation (GO:0030217) **(A)**, T cell activation (GO:0042110) **(B)** and T cell proliferation (GO:0042098) **(C)**. **(D)** Waterfall plot showing differentially expressed genes related to TH cell differentiation at FDR 0.1. Differentially expressed genes with an FDR < 0.1 are highlighted with a *. **(E, F)** Waterfall plots showing DEGs between Pou2af1^fl/fl^ x CD4-Cre and CD4-Cre control mice at FDR 0.05 related to IFN-γ production (GO:0032609) **(E)** and related to the response to IFN-γ (GO:0034341).

Significantly enriched GO terms at FDR 0.1 were identified related to molecular function. Top hits related to molecular function contained structural molecule activity (18), structural constituent of ribosome (16), translation regulator activity (10), rRNA binding (9), translation regulator activity/nucleic acid binding (8) and translation factor activity/RNA binding (8) (**Figure S4**). When narrowing analysis to T cell specific GO-terms, several genes associated with T cell differentiation (12), T cell activation (20) and T cell proliferation (11) at FDR 0.05 could be identified as differentially expressed in Pou2af1^fl/fl^ x CD4-Cre mice (**Figures 7A–C**). We also checked for the expression of key TFs and cytokines relevant for TH cell differentiation. *Tbx21* and *Infg* were downregulated (FDR < 0.1) in BOB.1/OBF.1-deficient CD4^+^ T cells confirming previous reports of reduced TH1 cytokines in conventional BOB.1/OBF.1-deficient mice and a direct contribution of BOB.1/OBF.1 to the *Infg* promoter (21) (**Figure 7D**). Consistently, three genes (*Pde4d, Itk, Irf8*) involved in IFN-γ production and seven genes (*Cd74*, *Ccl22, Ccl5, Irf8, Ifng, H2-Q7, Dapk1*) involved in the response to IFN-γ were differentially expressed at FDR < 0.05 (**Figures 7E, F**). *Rc3h1* and *Rc3h2* associated with negative regulation of TFH and TH17 cell differentiation were upregulated in BOB.1/OBF.1-deficient CD4^+^ T cells (FDR < 0.1) (**Figure S5A**). Interestingly, *Rc3h1* is also described as negative regulator of GC formation. However, the TFH cell signature gene *Bcl-6* was unaltered. In line with previous reports (24), *Zbtb32*, which encodes a transcriptional repressor of *Prdm1/Blimp1*, a known modulator of T cell memory, was downregulated (FDR < 0.1) (**Figure 7D**). Several genes associated with Treg cell differentiation or T cell function/differentiation during GC formation could also be identified as differentially expressed, but without statistical significance (**Figures S5B, C**).

Waterfall plot shows the top 30 DEGs in Pou2af1^fl/fl^ x CD4-Cre mice compared to CD4-Cre controls (**Figure 6C**). The strongest DEG was *2010007H06Rik*, which function is unknown so far. The most interesting genes among these top 30 DEGs regarding T cell signaling and function are summarized in **Table 1**. Interestingly in the context of BOB.1/OBF.1 being a transcriptional co-activator, four genes with DNA-binding TF activity (*Sox4, Ubn2, Irf8, Irf4*) were identified as differentially expressed in Pou2af1^fl/fl^ x CD4-Cre mice. Genes associated with chemokine signaling (*Ccr5, Ccl5, Ccl22*) necessary for chemoattraction of T lymphocytes were all downregulated in Pou2af1^fl/fl^ x CD4-Cre animals. Several genes associated with Treg, TFH and TH cell development (*Sox4, Kmt2a, Kmt2d, Irf8, Irf4, Cd74*) could be identified among the top 30 DEGs upon BOB.1/OBF.1 ablation specifically in CD4^+^ T cells. *In silico* analyses revealed consensus octamer motifs within the first 3,000 bp upstream of the transcriptional start site in eight genes that are among the top 30 DEGs in Pou2af1^fl/fl^ x CD4-Cre mice (*Rab4a, Sox4, Mid1, Fasl, Crppa, Mpeg1, Cep170, Dapk1*). Moreover, all other genes listed in **Table 1** reveal multiple putative Oct/BOB.1/OBF.1 binding sites in the promoter region that are either perfect or differ in one or two positions from the consensus site. However, all these sites harbor an adenine at position five of the octamer motif that is essential for ternary complex formation with Oct1 and BOB.1/OBF.1 (8, 44). Three representative possible BOB.1/OBF.1 binding sites for each gene are shown in **Figure S6**.

**Table 1:** Top DEGs in Pou2af1^fl/fl^ x CD4-Cre mice compared to CD4-Cre controls.

Gene	Gene Function	Regulation
**Rab4a**	Regulates CD4 surface expression	Upregulation
**Sox4**	TF, T cell differentiation, TFH cell differentiation	Upregulation
**Kmt2a**	TFH cell differentiation	Upregulation
**Kmt2d**	Treg development	Upregulation
**Irf4**	T helper cell activation and differentiation	Downregulation
**Fasl**	T cell apoptosis	Downregulation
**Mid1**	TCR signaling and migration of cytotoxic T cells	Downregulation
**Ccr5**	Lymphocyte migration	Downregulation
**Irf8**	TF, TFH cell regulation	Downregulation
**Ccl5**	Chemoattractant	Downregulation
**Ccl22**	Chemoattractant	Downregulation
**CD74**	T cell activation and differentiation	Downregulation

DEGs regarding T cell signaling and function from the top30 DEGs shown in waterfall plot of **Figure 6** are summarized here. All genes listed in Table 1 harbor multiple putative Oct/BOB.1/OBF.1 binding sites in the promoter region that are either perfect or differ in one or 2 positions from the consensus site.

## Discussion

The GC reaction is a complex process involving several immune cell subtypes, including B and T cells. Since BOB1/OBF.1 is expressed in both lymphocyte subsets, our study aimed to finally clarify whether and how BOB.1/OBF.1 expression in T cells contributes to the GC reaction. Therefore, the *in vivo* function of BOB.1/OBF.1 in distinct T cell subpopulations contributing to the GC reaction was studied applying conditional mutagenesis, allowing to investigate specifically BOB.1/OBF.1 function in CD4^+^ T or TFH cells, in the presence of immunocompetent B lymphocytes.

Our findings of an altered CD4/CD8 T cell ratio in Pou2af1^fl/fl^ x CD4-Cre mice characterized by significantly reduced levels of CD4^+^ T cells and at the same time increased levels of CD8^+^ T cells in the periphery provide evidence for a role of BOB.1/OBF.1 in CD4^+^ T cell biology. A reduced CD4^+^ T cell compartment was also described analyzing conventional BOB.1/OBF.1-deficient mice (21). Moreover, we found significantly reduced numbers of TFH cells when BOB.1/OBF.1 expression was deleted in a CD4- or IL21-Cre dependent manner. In particular, the reduction in Pou2af1^fl/fl^ x IL21-Cre mice shows that this is a TFH cell intrinsic defect and not exclusively a consequence of reduced CD4^+^ T cells prior to GC formation. Since IL21 is also required for TFH cell differentiation (38), crossing Pou2af1^fl/fl^ mice to IL21-Cre mice results in BOB.1/OBF.1 deficiency already in pre-TFH cells. Thus, BOB.1/OBF.1 might play a role already in pre-TFH cells and their subsequent differentiation. Our findings are contrary to the report by Yamashita et al., describing BOB.1/OBF.1 as a factor limiting TFH cell generation (31). However, it was already demonstrated that BOB.1/OBF.1 is highly expressed in TFH cells in mice and humans (22, 31, 45, 46). Reduced TFH cell numbers were found in a patient with a mutation in the *Pou2af1* gene leading to the absence of BOB.1/OBF.1 protein (27). Several studies also suggested an involvement of BOB.1/OBF.1 in TFH cell development. Chevnier et al. reported that BOB.1/OBF.1 deficiency prevents the differentiation of CD4^+^ T cells into TFH cells in an autoimmune mouse model (47). Moreover, BOB.1/OBF.1-deficient mice infected with influenza also featured reduced TFH cell numbers (23). However, when mice were reconstituted with BOB.1/OBF.1-deficient T and WT B cells prior to influenza infection TFH cells developed normally (23). Stauss et al. also reported a prominent reduction of TFH cells in BOB.1/OBF.1-deficient mice upon SRBC immunization and revealed a CD4^+^ T cell autonomous defect by performing reconstitution experiments (22). Moreover, loss of BOB.1/OBF.1 has been found to be associated with reduced Bcl6 expression in TFH cells from mixed BM chimeras. ChIP experiments of BOB-deficient CD4^+^ T cells maintained under TFH inducing conditions revealed binding of BOB.1/OBF.1 together with Oct-1 and Oct-2 to one of the six identified octamer motives within the *Bcl6* promoter (22). However, applying the here described genetic model we could not detect altered expression of *Bcl-6* in BOB.1/OBF.1-deficient CD4^+^ T cells by RNA-seq. In contrast to Stauss et al, we utilized total CD4^+^ T cells isolated after immunization for RNA-seq analysis instead of primary TFH or *in vitro* generated TFH cells, which might account for this discrepancy. Still, *Rc3h1* and *Rc3h2* associated with negative regulation of TFH differentiation were significantly upregulated in the present study. Additionally, *Cxcr5*, which was already shown to be cooperatively regulated by NF-κB, BOB.1/OBF.1 and Oct-2 in B cells, was slightly decreased on RNA level (48). Besides TFH cells, BOB.1/OBF.1 is also involved in the differentiation of further TH cell populations. We could already show that BOB.1/OBF.1 contributes to *Infg* and *IL-2* promoter activities in CD4^+^ T cells thereby controlling TH1/TH2 cytokine balance (21). Additionally, ChIP experiments showed binding of BOB.1/OBF.1 to the consensus octamer site of the *Infg* promoter (22). BOB.1/OBF.1-deficient TH2 cells revealed reduced levels of the transcriptional factor PU.1 resulting in higher GATA3 activity and consequently higher TH2 cytokine release (21). Mattes et al. confirmed this mechanism by showing that increased BOB.1/OBF.1 expression resulted in increased PU.1 levels and a suppression of GATA3 and subsequently TH2 cytokine production (49). However, expression of *Gata3* was not markedly changed in the here presented study addressed by RNA-seq analysis. Nevertheless, RNA-seq provided further evidence for a role of BOB.1/OBF.1 in TH1 cell differentiation. *Tbx21* and *Infg* were significantly downregulated in BOB.1/OBF.1-deficient CD4^+^ T cells and several genes involved in IFN-γ production and in the response to IFN-γ were significantly differentially expressed. Moreover, *Irf4* was significantly downregulated and was already shown to regulate TH1 responses as IRF4^-/-^ mice show reduced T-bet and IFN-γ expression (50). The role of IRF4 is certainly complex, as it is also involved in the regulation of TH2, TH9 and TH17 immune responses. As the T cell specific role of BOB.1/OBF.1 was controversially discussed in recent years, our approach of specific BOB.1/OBF.1 deletion in CD4^+^ T and TFH cells now underlines the requirement of BOB.1/OBF.1 for TH and especially TH1 and TFH cell differentiation.

More interestingly, when examining the response to SRBCs, we observed significantly reduced levels of GC B cells after both CD4^+^ T and TFH cell-specific BOB.1/OBF.1 deletion. These findings clearly indicate that the absence of GCs observed in conventional BOB.1/OBF.1-defcient mice cannot exclusively be traced back to the defective B cell compartment. Instead, our results provide evidence that BOB.1/OBF.1 expression in T cells is also required for efficient GC formation. The fact that the reduction of GCs is more prominent upon CD4^+^ T cell specific BOB.1/OBF.1 deletion suggests that BOB.1/OBF.1 is already required during GC initiation. This is in line with findings of *Pou2af1* being highly expressed in early stage GC-TFH cells (51). As a consequence of impaired GC formation both conditional knockouts also featured markedly reduced numbers of plasma cells and activated CD69^+^ B cells. Surprisingly, levels of total class-switched immunoglobulins were unaffected in Pou2af1^fl/fl^ x CD4-Cre mice. However, reduced titers of NP-specific antibodies were found suggesting impaired antigen-specific immune responses in these mice. Notably, higher proportions of Tregs were found in these animals. Two studies revealed an altered expression of *Pou2af1* in Tregs but so far, the function of BOB.1/OBF.1 in Tregs is unknown (52, 53). It was reported that Foxp3 can suppress *Pou2af1* expression in Tregs (54). Our RNA-seq analysis revealed a slight increase of FoxP3 expression and several other DEGs associated with Treg cell differentiation. Moreover, the histone methyltransferase Kmt2d, which was among the top 30 DEGs was shown to bind to FoxP3 enhancer elements thereby enabling TF binding at genes involved in Treg cell differentiation (55). In conclusion, the question whether BOB.1/OBF.1 is also involved in Treg physiology and therefore in regulating the balance of induction and suppression of an ongoing GC reaction remains to be elucidated.

In addition, RNA-seq analysis revealed further genes associated with T cell differentiation and activation among the top 30 DEGs. Genes necessary for chemoattraction of T lymphocytes (*Ccr5, Ccl5, Ccl22*) were all downregulated in Pou2af1^fl/fl^ x CD4-Cre animals and revealed possible BOB.1/OBF.1 binding sites in the promoter region, which supports our findings of reduced CD4^+^ T cell numbers in spleen and LNs. The *Ccr5* gene was already found to be regulated by BOB.1/OBF.1 and Oct-1 in T cells (56). Eight genes with consensus octamer motives could be identified among the top 30 DEGs. Among them, small GTPase Rab4a which is involved in protein transport and vesicular trafficking. Rab4a is known to regulate surface expression of CD4 *via* endosomal recycling and was shown to cause pro-inflammatory T cell lineage specification and to trigger autoimmunity (57). Sox4 is a transcriptional activator that binds with high affinity to the T cell enhancer motif 5’-AACAAAG-3’ and is involved in T cell differentiation in the thymus (58). Its role in peripheral T cells and immune responses is incompletely understood. It was shown that Sox4 can be induced by TGF-β and negatively regulates the TF GATA-3, the master regulator of TH2 cells (59). Recently, increased expression of Sox4 in follicular regulatory innate lymphoid cells was identified as one mechanism to inhibit GC-TFH and GC-B cell interaction (60). Mid1 is a microtubule-associated ubiquitin ligase regulating protein phosphatase 2A activity (61). Mid1 was shown to be upregulated in cytotoxic lymphocytes controlling TCR signaling and exocytosis of lytic granules, but a function in CD4^+^ T cells was not described to date (62). The expression of FasL is involved in the regulation of apoptotic and anti-apoptotic signaling pathways in T cells and FasL expression by T cells is often associated with their activation (63). No T cell related function is described for Crppa, Mpeg1, Cep170 and Dapk1, which also revealed consensus octamer motives. Moreover, a requirement of BOB.1/OBF.1 for the generation and function of CD4^+^ memory T cells was described (24). Shakya et al. demonstrated that BOB.1/OBF.1 and Oct-1 localize the histone lysine demethylase Jmjd1a to targets like *Il2, Ifng* and *Zbtb32* thereby contributing to CD4^+^ T cell memory responses (24). Consistently, we also identified *Zbtb32* as significantly downregulated in BOB.1/OBF.1-deficient CD4^+^ T cells. Unfortunately, *Il2* expression could not be addressed as it was not analyzed by RNA-seq. The identification of *Icosl* among the top 30 DEGs is surprising, since Icosl is known to be expressed on APCs, but not on T cells. This observation might be caused by contamination with non- T cells as cells were isolated by magnetic separation. The overall purity was ≥ 96%, but there is one sample in the group of CD4-Cre controls which revealed slightly less purity than the other samples. Due to the low sample size in the CD4-Cre control group this might account for the differences we observe in *Icosl* expression. However, our observation might also support earlier findings by Wang et al., who were able to detect Icosl on activated T cells with their generated and verified anti-Icosl antibody (64). Moreover, they confirmed that *Icosl* transcription is induced in activated CD4^+^ T cells by qRT-PCR. Also, other B7 family molecules, typically expressed on APCs, have been shown to be expressed also on activated T cells indicating that T cells could act as APCs to trigger the activation of neighboring or resting T cells (65). To finally verify these observations of Icosl expression by activated T cells, further investigations are required. Altogether, several possible BOB.1/OBF.1 regulated genes that are interesting players in T cell physiology could be identified. However, it is difficult to draw clear conclusions about the role of BOB.1/OBF.1 for their function, as most of the identified DEGs are involved in the regulation of many different partly opposing processes during T cell differentiation and function.

Collectively, we provide evidence that BOB.1/OBF.1 is essential in CD4^+^ T cell biology. Our approach of specific BOB.1/OBF.1 deletion in CD4^+^ T and TFH cells underlines the requirement of BOB.1/OBF.1 for TFH cell development and GC formation. RNA-seq analysis identified several possible BOB.1/OBF.1 target genes important for T cell differentiation.

## Data Availability Statement

The datasets presented in this study can be found in online repositories. The names of the repository/repositories and accession number(s) can be found below:https://www.ncbi.nlm.nih.gov/sra/?term=PRJNA814427, accession ID: PRJNA814427.

## Ethics Statement

The animal study was reviewed and approved by Regierungspräsidium Tübingen. Written informed consent was obtained from the owners for the participation of their animals in this study.

## Author Contributions

AB performed experiments, analyzed the data, and wrote the manuscript. JE and TK analyzed RNA-seq data. TH and TW provided advice and helped drafting the manuscript. CB designed and supervised the study and helped drafting the manuscript. All authors contributed to the article and approved the submitted version.

## Funding

This work was supported by a grant from the German Research Foundation (DFG) to CB (BR 2891/8-1).

## Conflict of Interest

The authors declare that the research was conducted in the absence of any commercial or financial relationships that could be construed as a potential conflict of interest.

## Publisher’s Note

All claims expressed in this article are solely those of the authors and do not necessarily represent those of their affiliated organizations, or those of the publisher, the editors and the reviewers. Any product that may be evaluated in this article, or claim that may be made by its manufacturer, is not guaranteed or endorsed by the publisher.

## Abbreviations

DEG, differentially expressed gene; GC, Germinal Center; LN, lymph node; Pou2af1; POU domain class 2-associating factor 1; SRBC, sheep red blood cell; TF, transcription factor; TFH, follicular T helper cells; TH cell, T-helper cell; Treg, regulatory T cells.
