# Supplementary material for: T Cell Specific BOB.1/OBF.1 Expression Promotes Germinal Center Response and T Helper Cell Differentiation
Source: Front Immunol. 2022 May 4;13:889564. doi: 10.3389/fimmu.2022.889564 (PMC9114770; doi:10.3389/fimmu.2022.889564)
Supplement: Supplementary file 1 [file DataSheet_1.pdf]

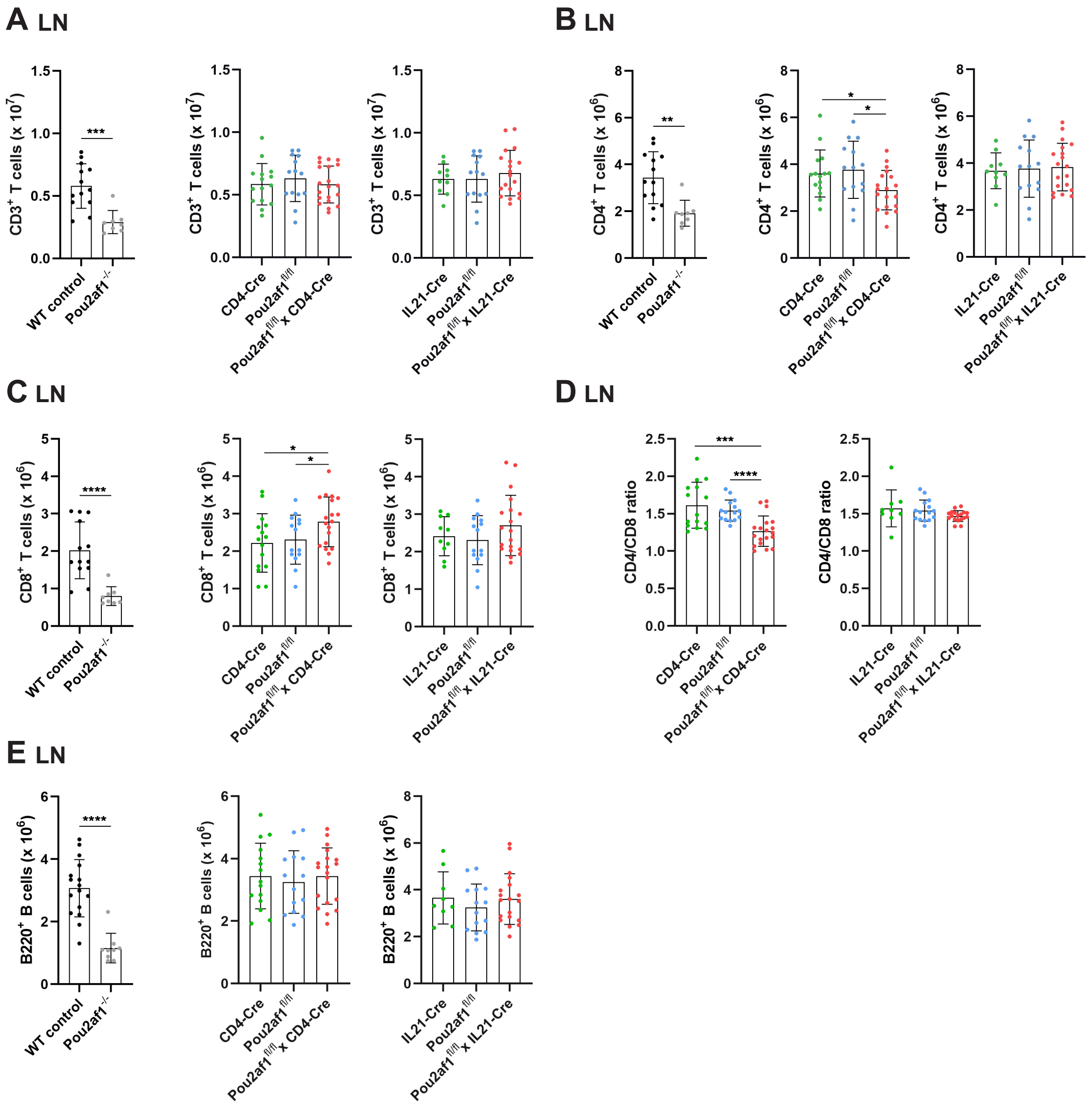

Fig. S1: BOB.1/OBF.1 deficiency in CD4$^+$ T cells alters the CD4/CD8 T cell ratio in the periphery. (A – C) Statistical representation of CD3$^+$ (A), CD4$^+$ (B) and CD8$^+$ (C) T cell numbers in the LN assessed by flow cytometry. Each point represents data from a single mouse. Data in the graphs are shown as means ± SD (n= at least 13 mice per group). (D) Statstical analysis of the CD4/CD8 T cell ratio in the LN. Gating strategy is shown in Fig S8 (E) Statistics of flow cytometric analyses showing B220$^+$ B cell numbers in the LN. Data are merged from at least three independent experiments. * $p < 0.05$, *** $p < 0.001$, and **** $p < 0.0001$. P-values were determined using a two-tailed Student's t test or Mann-Whitney-U test.

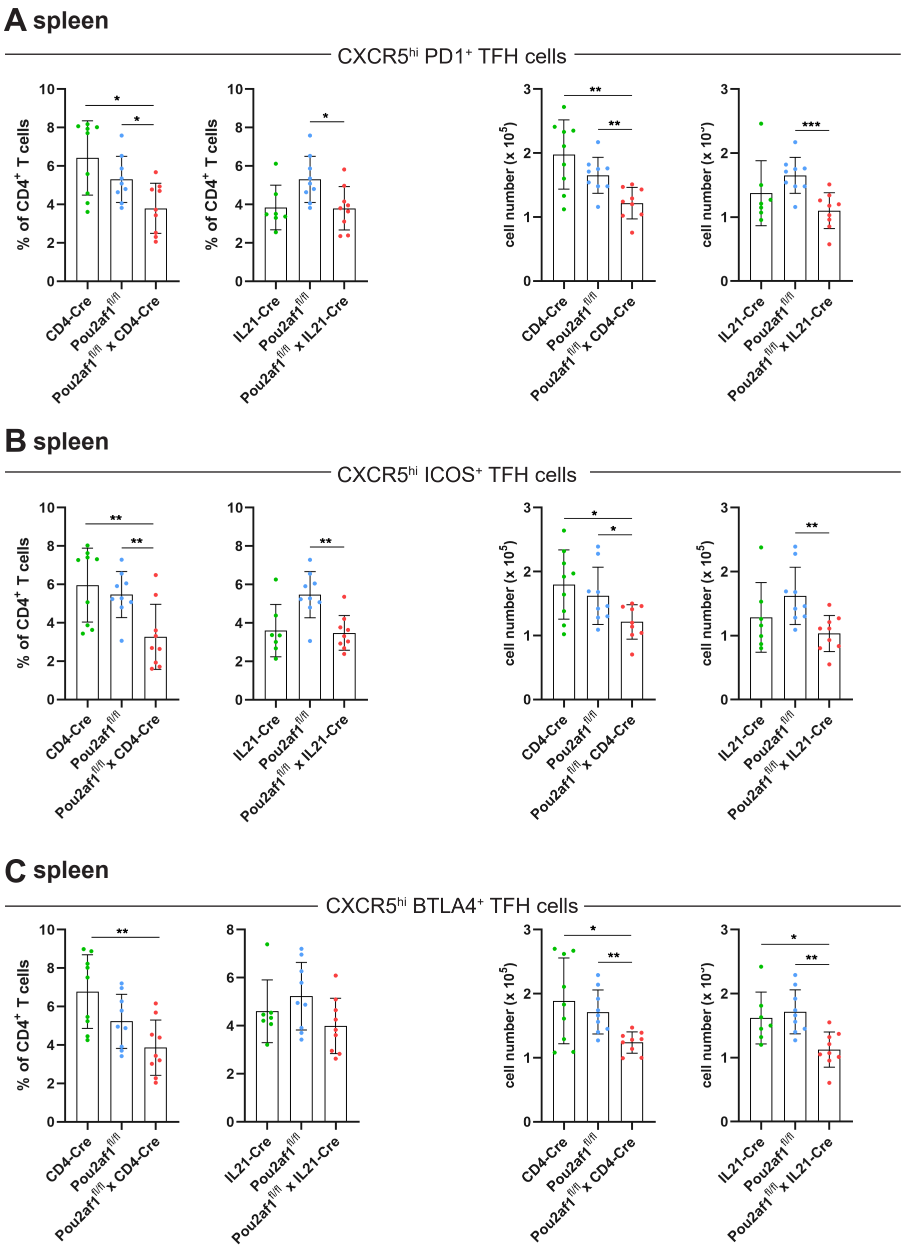

Fig. S2: BOB.1/OBF.1 is required for TFH cell development. (A - C) Statistical analyses of CXCR5^{high} PD1^{+} (A), CXCR5^{high} ICOS^{+} (B) and CXCR5^{high} BTLA4^{+} (C) TFH cell percentages and numbers of the LN are shown. Gating strategy is shown in Fig. S10. Each point represents data from a single mouse. Data in the graphs are shown as means ± SD (n= 9 mice per group). Data are merged from two independent experiments. * $p < 0.05$, ** $p < 0.01$, *** $p < 0.001$, and **** $p < 0.0001$. P-values were determined using a two-tailed Student's t test or Mann-Whitney-U test.

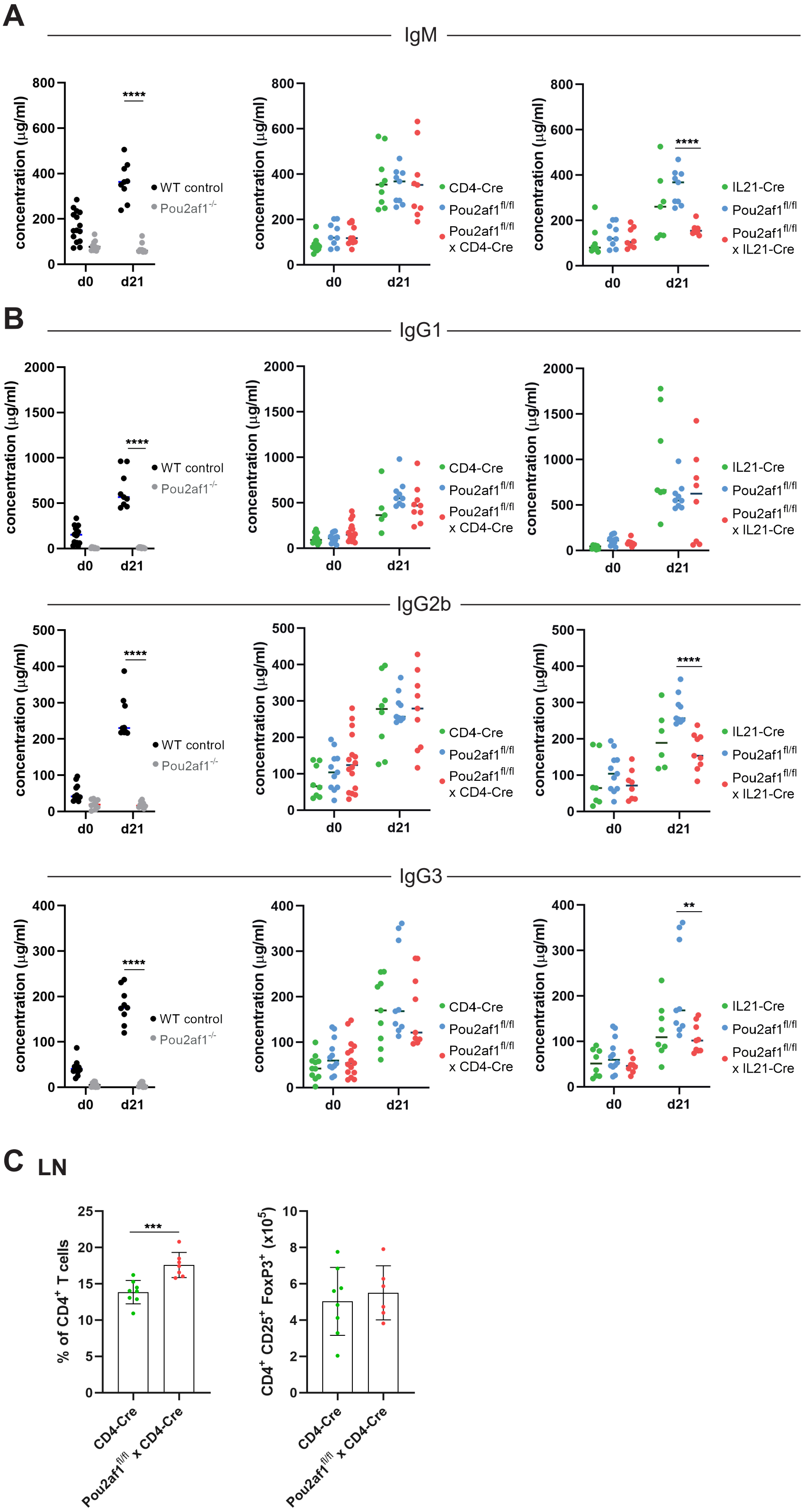

Fig. S3: Immunoglobulin levels after SRBC immunization. (A) Statistical representation of IgM serum concentrations (µg/ml) determined relative to a standard IgM. (B) Statistical representation of IgG1, IgG2b and IgG3 serum concentrations (µg/ml) determined relative to corresponding standard antibodies. Sera were measured at three different dilutions with technical triplicates each. (C) Statistical analysis of Treg cell percentages and number of the LN is shown. Each point represents data from a single mouse. Data in the graphs are shown as means. Data are merged from two independent experiments. ** $p < 0.01$, *** $p < 0.001$ and **** $p < 0.0001$. P-values were determined using a two-tailed Student's t test or Mann-Whitney-U test.

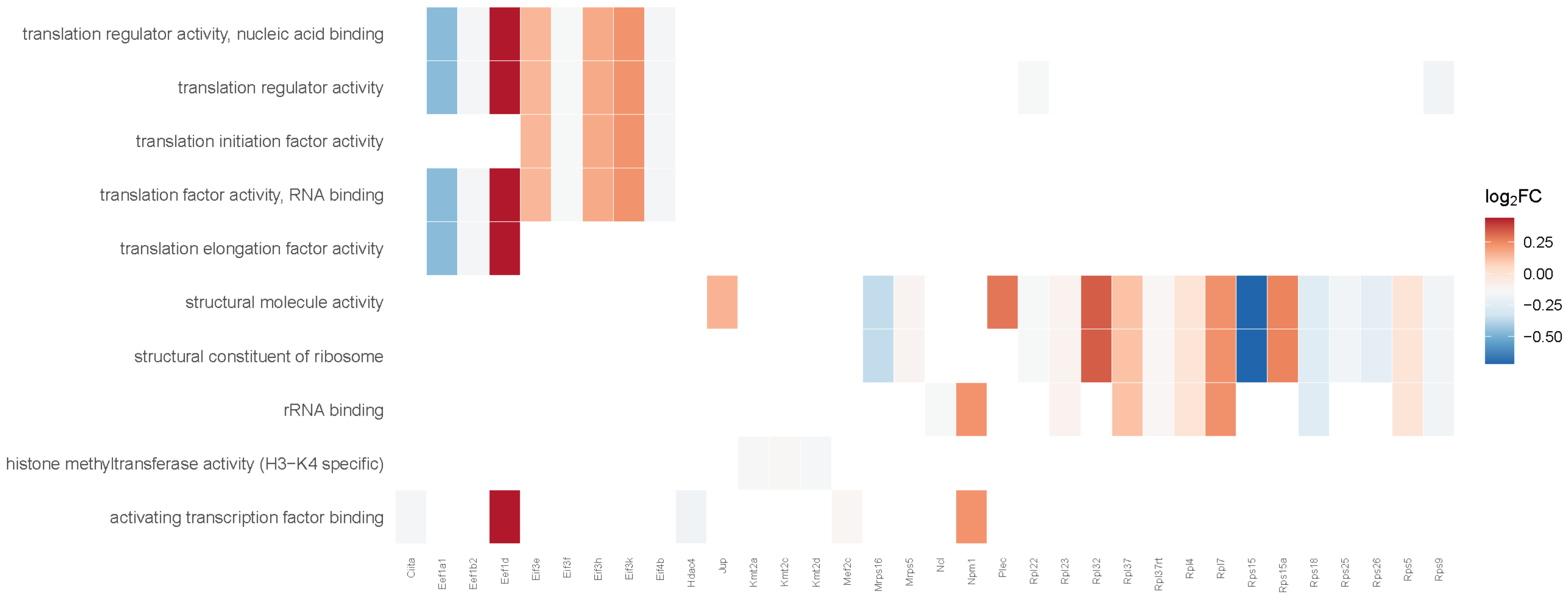

Fig. S4: Heatplot showing significantly enriched GO-terms related to molecular function. GO pathways were considered significant at FDR < 0.1.

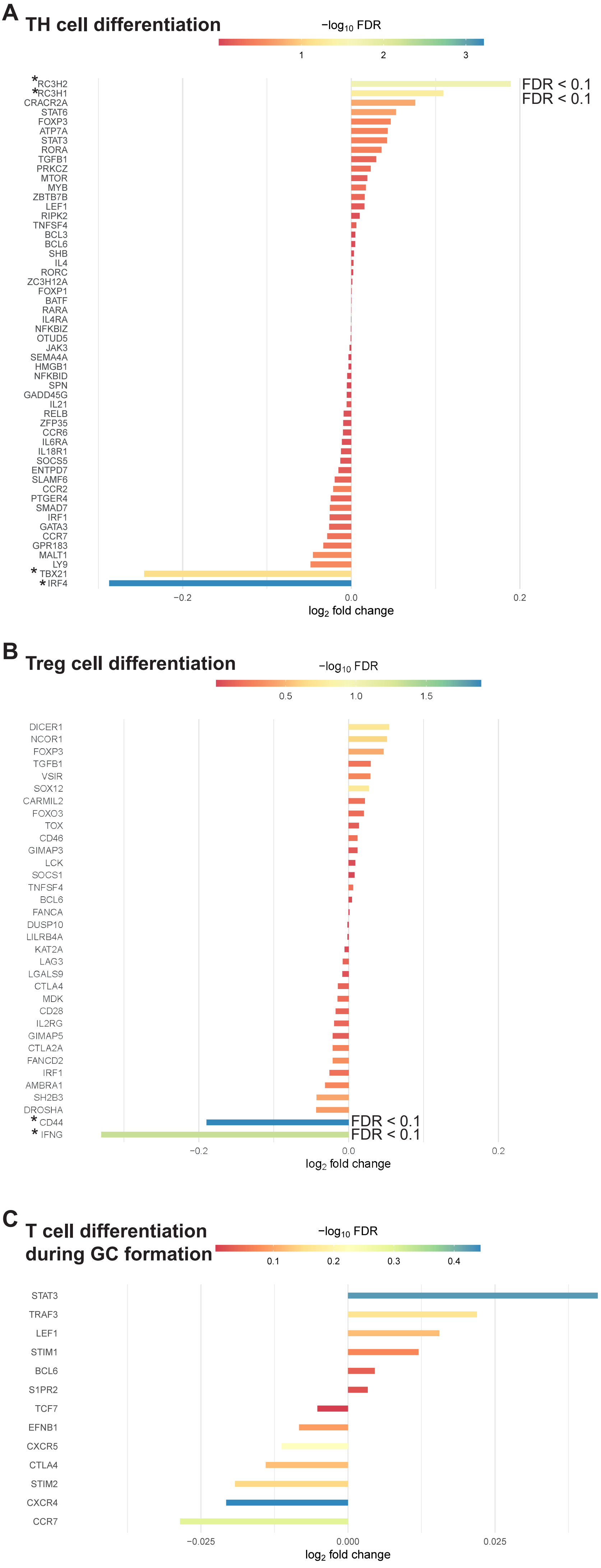

Fig. S5: Differential gene expression between CD4$^+$ T cells of Pou2af1$^{fl/fl}$ x CD4-Cre and CD4-Cre control mice (A - C) Waterfall plots showing DEGs at FDR 0.1 related to T helper cell differentiation (GO:0042093) (A) Treg cell differentiation (GO:0045066) (B) and genes related to T cell differentiation during GC formation. Significant DEGs with an FDR < 0.1 are highlighted with a *.

A

Rab4a M1	GAACATTACAGATTTGCATCTGGGAAAGAA
Rab4a M2	TCAACAGCAAACTTTGCATGCTGTGCCAGC
Rab4a M3	TCCCGACCCCAATTTGCCTTTCAATAAATG
Sox4 M1	CTAGAAGTGGCATTTGCATTAGGGAGAACT
Sox4 M2	GGGCTTTCGATTACGTTTCACCACCGGTGA
Sox4 M3	AAGAAAAGAGATTTTGCACCAAAGGCTGAT
Mid1 M1	TACATGTAATCATGCAAATTCACAAACCTT
Mid1 M2	CTTTGTGATCAGTTTGCATTGGTGCTGGCT
Mid1 M3	TTTCACCAGGAATGCAATTCATGTTTACCT
Fasl M1	AGATTAAATATATGCAAATAGTTGTTTTTG
Fasl M2	TATTTTTAAATATGCACATGCATGTGTGTC
Fasl M3	GACTGAGCTGCTAAACGAGCATCCTGGAGG
Crppa M1	GGGTTGTTTTGATTTGCATTTCCCTGATGA
Crppa M2	CCTTCTACTCTGTGCAAATGGCATGACATG
Crppa M3	ACCCAAACCCAAAGCAAAGCTAGGGCATGC
Mpeg1 M1	CCTGTCCTGGCATGCAAATGGAGGTTGTGC
Mpeg1 M2	GATGAAAAAGTTAAACGTGTGGTCAGTTGC
Mpeg1 M3	TAAACCAAACTTTGCAAATTTGTAGTGTGG
Cep170 M1	ACTACACCAGAATTTGCATGTGTAAGAAAT
Cep170 M2	CGCCCTTTCCCTTTTGCATCGCTTGGATTA
Cep170 M3	AATTTTAAAGGAAGCAAATGCTAGCAGCTA
Dapk1 M1	CAGATGTATTTATTTGCATGCTTCCCAAGT
Dapk1 M2	GTTTTCAAAGTTACGTTTCCTTTAGCTTTT
Dapk1 M3	TATTCAGAAGACACGTTTCTGTGATCACTT

B

Kmt2a M1	GAGCCTTGCCATTTTGCATAGAATCAATTG
Kmt2a M2	TGAAGGCCGTTATGCATATTAAAGAAGGGG
Kmt2a M3	AGCCTCAGCCAAGGCAAATTCTTCGGGCAG
Kmt2d M1	TTCCTCTCCGGTTGCAAATGGCTGCCTCGT
Kmt2d M2	TGGGGTTCCCCAGGCAAAAGGGCCCCCTGG
Kmt2d M3	CTTCCGGCCCCCAAACGTGGCAAGAACCCA
Irf4 M1	TCCAGTATCCCCTTTGCATGCCTCCACCTT
Irf4 M2	CGGGGTATGCTGTTTGCAAGGAACGGTTGA
Irf4 M3	ATCACTGCTGTGTGCAACTAATAGATGCTA
Ccr5 M1	CATAAGTGCCTATGCAAAATGTGTAATTTT
Ccr5 M2	ATGAAATTCGCAGGCAAATGGATGGAACTT
Ccr5 M3	TCTTGAACAGGATTTGCAATGGAATTTCAG
Irf8 M1	GGAAAATCCTTATTTGAATTCGTATTTATT
Irf8 M2	GGGTCGGGGACGTGCAAAAGTGATTTCTCG
Irf8 M3	TCATGGCCAAGAAGCAAGTTGGGGAGGAAA
Ccl5 M1	CTACATTCTGAATGCAAACACAGAGATGGG
Ccl5 M2	TCAGGATTACCTGGCAAATTCCTTACAACA
Ccl5 M3	ACAACTTGGGAATTTGCCAAGTGAAGACCA
Ccl22 M1	GCAGCATGGGGAGGCAAATCTACTGGCGTC
Ccl22 M2	TATACACAAGTATGCAATTGACACTATAGT
Ccl22 M3	TGGAGCTTTGAGTTTGCACACTCACTCTGG
CD74 M1	GGTAACTAAGCATTCAAATATATGAGTCTA
CD74 M2	GGTGCGTTGGGAAGCAAACAAACAAGATTT
CD74 M3	CAACCATCTCTGCTTGCATGTATGAGCCTG

Fig. S6: Identification of possible BOB.1/OBF.1 binding sites in the promoters of DEGs. *In silico* search for potential Oct1/2 and BOB.1/OBF.1 binding sites within the first 3.000 bp upstream of the transcriptional start site of the *Rab4a, Sox4, Mid1, Fasl, Crppa, Mpeg1, Cep170* and *Dapk1* (A) or *Kmt2a, Kmt2d, Irf4, Ccr5, Irf8, Ccl5, Ccl22* and *CD74* (B) genes. Possible binding sites are marked in red. Nucleotides that differ from the consensus octamer sequence ATGCAAAT are marked in blue.

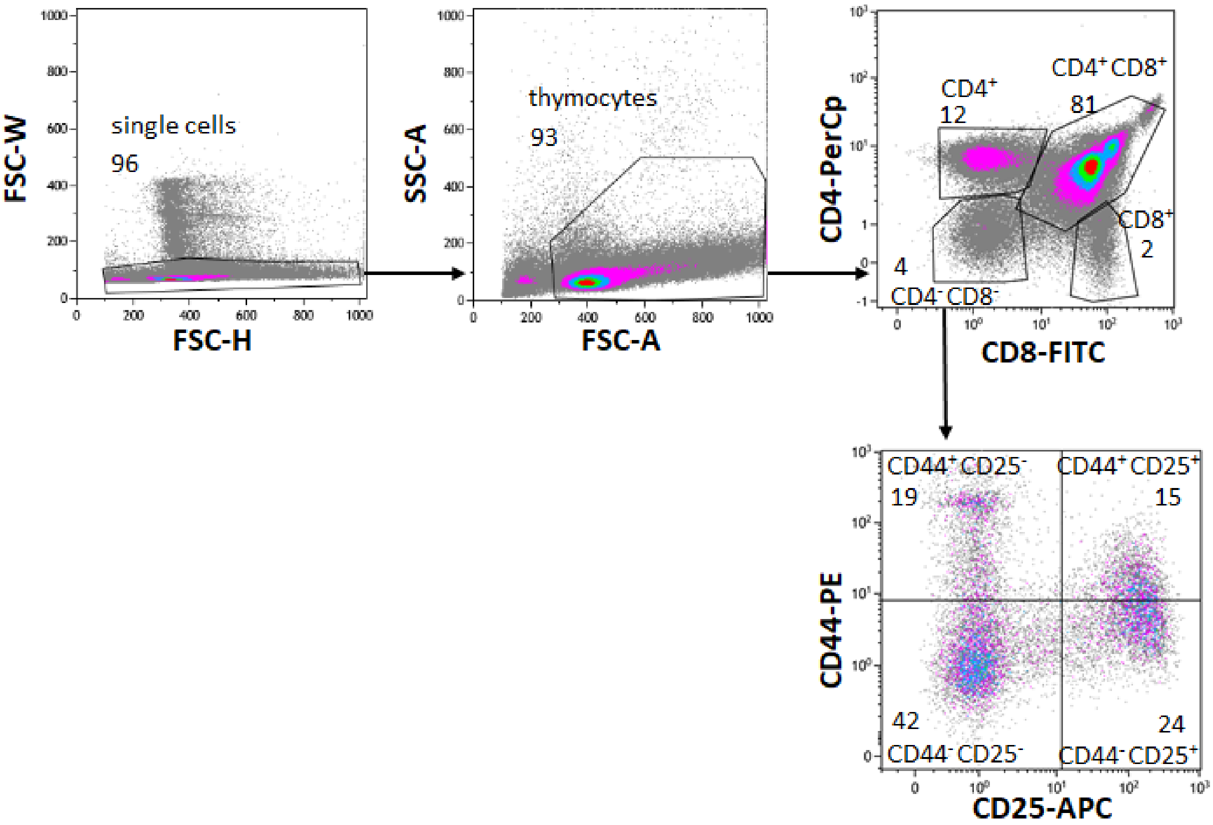

Fig. S7: Gating strategy for the identification of CD4/CD8 and CD44/CD25 thymocytes. A single cell suspension was prepared from thymocytes of mice and stained with fluorochrome-conjugated antibodies against CD4 (PerCP), CD8 (FITC), CD44 (PE) and CD25 (APC). Data were acquired on a Gallios cytometer (Beckman Coulter) and analyzed with Kaluza Analysis software, version 2.1 (Beckman Coulter). Thymocytes were identified by their scatter properties (FSC-A x SSC-A plot).

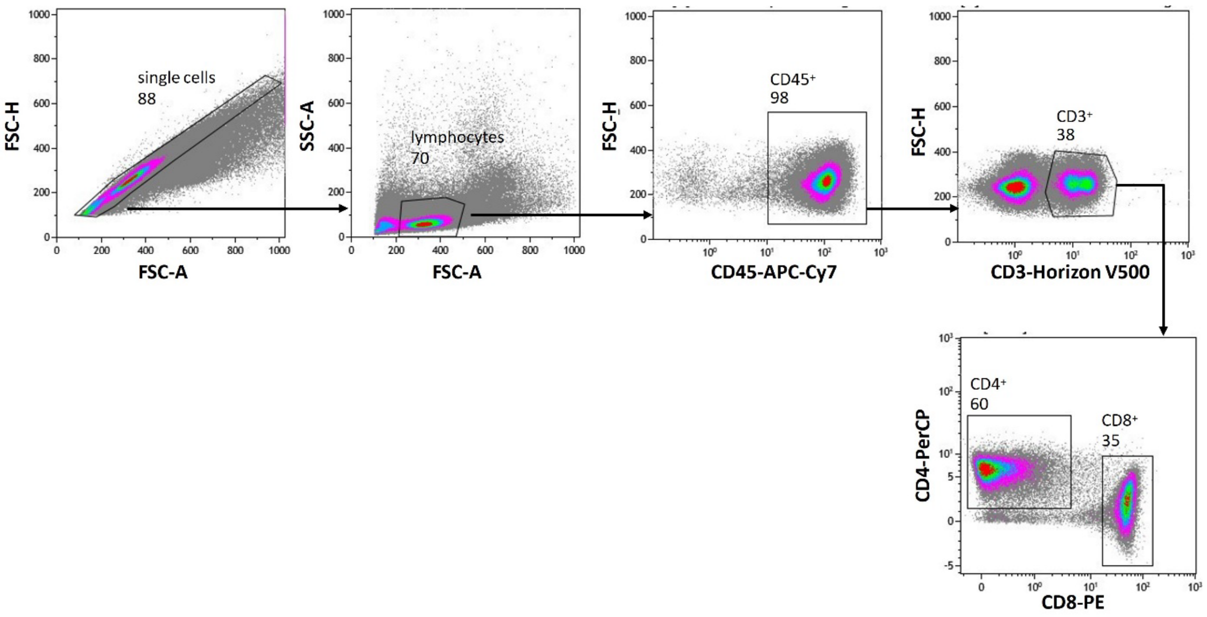

Fig. S8: Gating strategy for the identification of $CD4^+$ and $CD8^+$ T cells. A single cell suspension was prepared from spleens and LNs of mice and stained with fluorochrome-conjugated antibodies against CD45 (APC-Cy7), CD3 (V500), CD4 (PerCP) and CD8 (PE). Data were acquired on a Gallios cytometer (Beckman Coulter) and analyzed with Kaluza Analysis software, version 2.1 (Beckman Coulter). Lymphocytes were identified by their scatter properties (FSC-A x SSC-A plot).

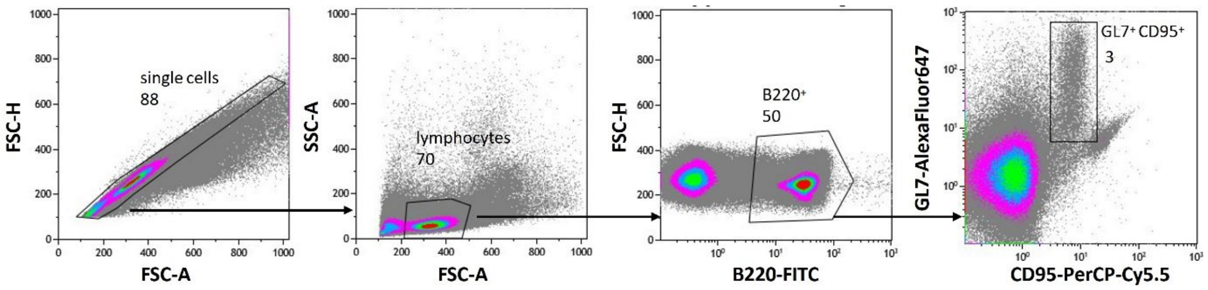

Fig. S9: Gating strategy for the identification of GL7$^+$ CD95$^+$ GC B cells. A single cell suspension was prepared from spleens and LNS of mice and stained with fluorochrome-conjugated antibodies against B220 (FITC), GL7 (AF647) and CD95(PerCP-Cy5.5). Data were acquired on a Gallios cytometer (Beckman Coulter) and analyzed with Kaluza Analysis software, version 2.1 (Beckman Coulter). Lymphocytes were identified by their scatter properties (FSC-A x SSC-A plot).

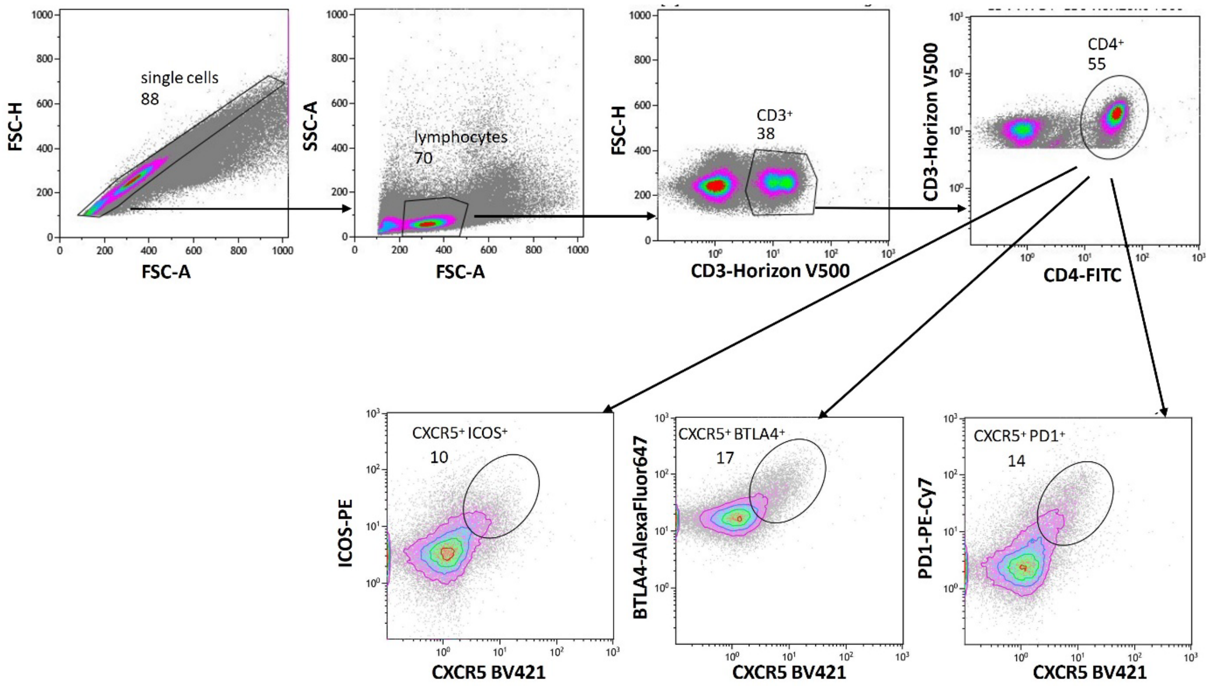

Fig. S10: Gating strategy for the identification of CXCR5$^+$ PD1$^+$, CXCR5$^+$ ICOS$^+$, CXCR5$^+$ Btla4$^+$TFH cells. A single cell suspension was prepared from spleens of mice and stained with fluorochrome-conjugated antibodies against CD3 (V500), CD4 (FITC), CXCR5 (BV421), ICOS (PE), PD1 (PE-Cy7) and Btla4 (AF647). Data were acquired on a Gallios cytometer (Beckman Coulter) and analyzed with Kaluza Analysis software, version 2.1 (Beckman Coulter). Lymphocytes were identified by their scatter properties (FSC-A x SSC-A plot).

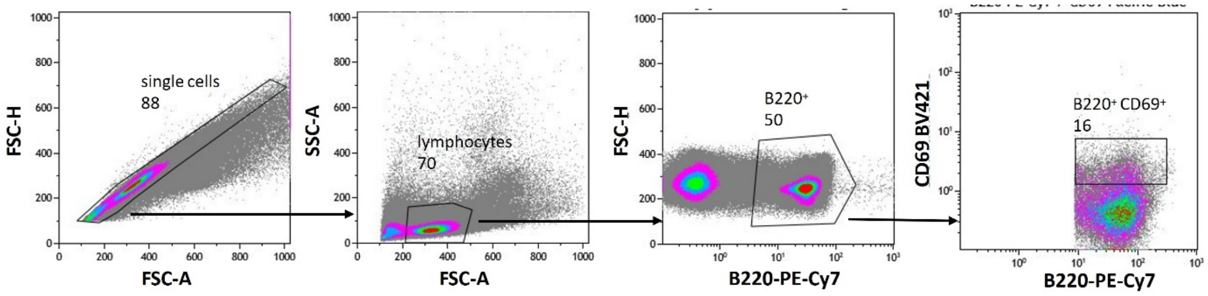

Fig. S11: Gating strategy for the identification of CD69$^+$ B cells. A single cell suspension was prepared from spleens and LNS of mice and stained with fluorochrome-conjugated antibodies against B220 (PE-Cy7) and CD69 (BV421). Data were acquired on a Gallios cytometer (Beckman Coulter) and analyzed with Kaluza Analysis software, version 2.1 (Beckman Coulter). Lymphocytes were identified by their scatter properties (FSC-A x SSC-A plot).

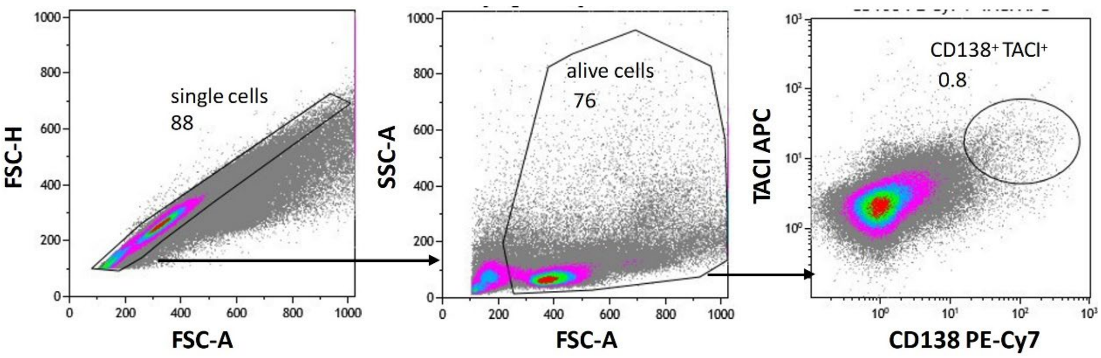

Fig. S12: Gating strategy for the identification of CD138$^+$ TACI$^+$ plasma cells. A single cell suspension was prepared from spleens and LNS of mice and stained with fluorochrome-conjugated antibodies against CD138 (PE-Cy7) and TACI (APC). Data were acquired on a Gallios cytometer (Beckman Coulter) and analyzed with Kaluza Analysis software, version 2.1 (Beckman Coulter). Lymphocytes were identified by their scatter properties (FSC-A x SSC-A plot).

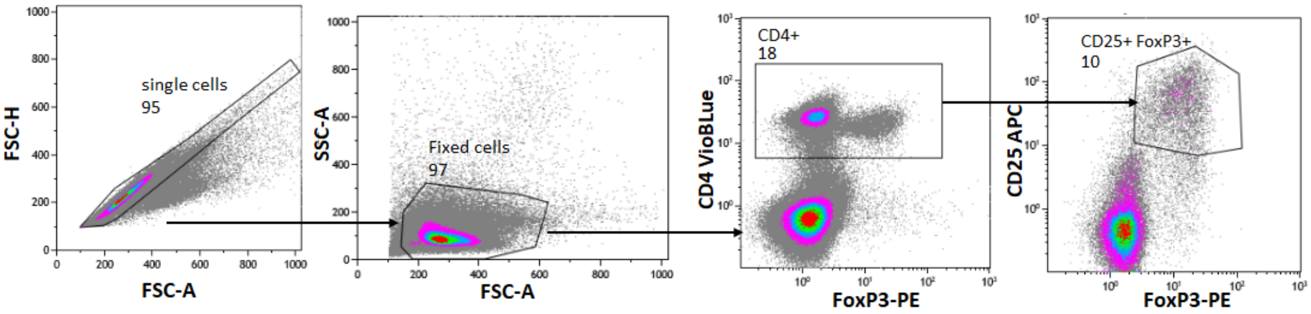

Fig. S13: Gating strategy for the identification of CD4$^+$ CD25$^+$ FoxP3$^+$ Tregs. A single cell suspension was prepared from spleens and LNS of mice and stained using Treg detection kit (Milteny Biotec). Data were acquired on a Gallios cytometer (Beckman Coulter) and analyzed with Kaluza Analysis software, version 2.1 (Beckman Coulter). Lymphocytes were identified by their scatter properties (FSC-A x SSC-A plot).

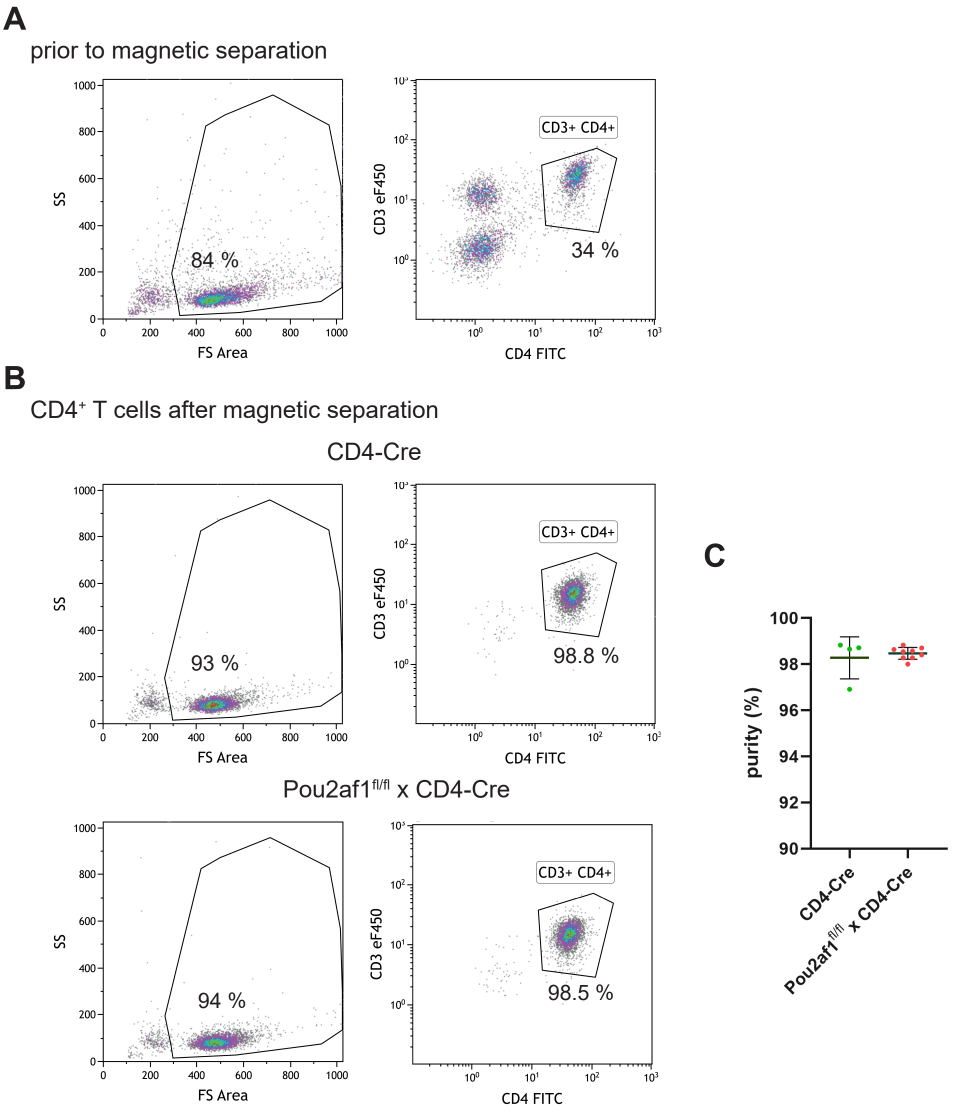

Fig. S14: CD4$^+$ T cells were isolated from LNs of CD4-Cre (n=4) and Pou2af1$^{fl/fl}$ x CD4-Cre (n=9) mice after immunization with SRBC by magnetic separation. (A) Respective FSC/SSC gate on lymphocytes and CD3$^+$ CD4$^+$ T cells prior to magnetic separation are shown. (B) Respective FSC/SSC gate on lymphocytes and purity of isolated CD3$^+$ CD4$^+$ T cells after magnetic separation of CD4-Cre (upper panel) and Pou2af1$^{fl/fl}$ x CD4-Cre (lower panel) is shown. (C) Overview of purity of isolated CD3$^+$ CD4$^+$ T cells of all analyzed mice.

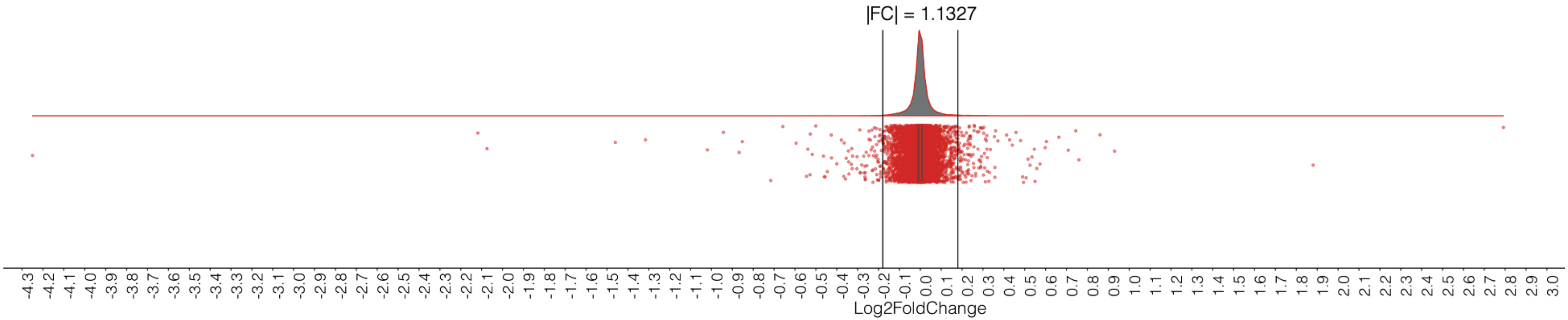

Fig. S15: Distribution of log2foldchanges. The 99th percentile of the log2foldchanges was calculated and applied as a cutoff to outline only the most prominently regulated genes. This cutoff corresponds to an absolute foldchange of 1.1327.

Table S1: Information of used flow cytometry antibodies

Antibody	Source	Identifier
anti-B220 FITC	BD	Cat #553088
anti-B220 PE-Cy7	eBioscience	Cat #12-0452-83
anti-BTLA4 APC	eBioscience	Cat #17-5956-82
anti-CD138 PE-Cy7	Biolegend	Cat #142514
anti-CD25 APC	eBioscience	Cat #17-0251-82
Anti-CD25 APC	Milteny Biotec	Cat #130-120-674
anti-CD3 V500	BD	Cat #560771
anti-CD4 FITC	BD	Cat #553651
anti-CD4 PerCP	BD	Cat #553052
Anti-CD4 VioBlue	Miltenyi Biotech	Cat #130-120-674
anti-CD44-PE	Biolegend	Cat #103023
anti-CD45 APC-Cy7	BD	Cat #557659
anti-CD69 BV421	Biolegend	Cat #104528
anti-CD8 FITC	eBioscience	Cat #11-0081-82
anti-CD8 PE	Biolegend	Cat #100708
anti-CD95 PerCP-Cy5.5	Biolegend	Cat #152610
anti-CXCR5 BV421	BD	Cat #562889
anti-GL7 AlexaFluor 647	BD	Cat #561529
anti-ICOS PE	eBioscience	Cat #12-9942-82
anti-PD1 PE-Cy7	Biolegend	Cat #109110
anti-TACI APC	eBioscience	Cat #17-5942-82
Anti-FoxP3 PE	Miltenyi Biotech	Cat #130-120-674

Table S2: DEGs in Pou2af1$^{fl/fl}$ x CD4-Cre mice compared to CD4-Cre controls analyzed by RNA-seq

GeneID	log2FoldChange	pvalue	padj	sig	AbsFolds
Pou2af1	-4.25000768471932	2.576066155319e-62	3.45270146797405e-58	Downregulated	19.0274151919936
2010007H06Rik	2.79141148316464	1.09408190871722e-45	7.33198991126842e-42	Upregulated	6.92306782941018
Rab4a	1.88056208163635	6.97710154269354e-12	3.11713639922405e-08	Upregulated	3.68218492183873
Osbpl8	0.194830723534183	6.50693074684787e-09	2.18030982000005e-05	Upregulated	1.14458985398013
Rps27rt	-0.246388201470661	1.90353200446448e-08	4.25217324263958e-05	Downregulated	1.18623364911627
Wdfy1	0.335554160070951	8.685489970727e-08	0.000166302317253791	Upregulated	1.26186201523851
Prag1	-0.256034302836351	1.32322540995591e-07	0.00017735190169639	Downregulated	1.19419158092791
Spag9	0.230815536055403	2.52975115168646e-07	0.000243011368050969	Upregulated	1.17349812444021
Dapk1	0.308074791914204	2.45847740015975e-07	0.000243011368050969	Upregulated	1.23805447215165
Dapl1	-0.303382212875554	2.7615515957759e-07	0.000246753840254563	Downregulated	1.23403405887437
Ptprf	0.604226887673844	3.55506021131849e-07	0.000280285129484127	Upregulated	1.52016390853907
Plec	0.228222139621908	4.45090331934895e-07	0.000331419206606855	Upregulated	1.17139053272107
Celf1	0.194745977098684	1.30687114317328e-06	0.000612534410607978	Upregulated	1.14452262074056
Irf4	-0.28720826514	1.32533745487065e-06	0.000612534410607978	Downregulated	1.2202766547368
Vmn2r97	0.356774102303628	1.28536859181116e-06	0.000612534410607978	Upregulated	1.28055933520034
Mir703	-0.196408055377831	3.35563791827781e-06	0.00101117320992405	Downregulated	1.14584194487591
Lnpep	0.217600835509559	3.46762117982887e-06	0.00101117320992405	Upregulated	1.16279827431114
Ccl5	-0.715991901958136	4.05103891324544e-06	0.00113116821987976	Downregulated	1.64261218127452
2810455O05Rik	0.597619045752111	4.17506013210031e-06	0.00113976064252808	Upregulated	1.51321716407567
Pde4d	0.237235693462195	4.36242632268931e-06	0.00114646274515696	Upregulated	1.17873196182811
Dennd5a	0.227754886335003	4.68675539288857e-06	0.00116168611851666	Upregulated	1.1710112096927
Jup	0.572662514783996	4.55206260172507e-06	0.00116168611851666	Upregulated	1.48726581021934
Adcy6	0.289764168645421	4.88951136981494e-06	0.00116194151827487	Upregulated	1.22244043410812
Cep170	0.306969716727706	5.73818037947324e-06	0.00122983906959605	Upregulated	1.23710651060282
Plek	-1.31601847739879	5.80700717539882e-06	0.00122983906959605	Downregulated	2.48978035856369
Tet3	0.196971353851786	6.54880739049514e-06	0.0012907891978648	Upregulated	1.14628942478318
Irf8	-0.544588373952449	6.9261141302024e-06	0.00132615296695861	Downregulated	1.45860411967984
Sox4	0.44063110661117	7.17972547636107e-06	0.00133652584110649	Upregulated	1.3571979035166
Mpeg1	-1.46025996354582	8.57780464671491e-06	0.00149309500883013	Downregulated	2.75157940695581
Srgn	-0.23935246288273	9.04539056383343e-06	0.00155429961188538	Downregulated	1.18046270536405
Gm6477	-0.201906984568797	1.31651186732987e-05	0.00206438293685562	Downregulated	1.15021773253051
Ubn2	0.303343792176426	1.34036770804661e-05	0.00206493659666078	Upregulated	1.23400119550503
Mid1	-0.46363457834485	1.43726176114091e-05	0.00214040215384129	Downregulated	1.37901158637726
Rev3l	0.230317514350901	1.45396563415116e-05	0.00214148366972836	Upregulated	1.17309310005298
Mrps5	-0.199249890061998	1.57339177480861e-05	0.00226754515674837	Downregulated	1.1481012599322
Cep85	-0.265279312838821	1.98214648334893e-05	0.00268350599154806	Downregulated	1.20186871468397
Epsti1	-0.235522354443256	2.65549538175247e-05	0.0034554955923911	Downregulated	1.17733293523883
Wscd2	-0.328103419144976	2.89856724018075e-05	0.00371051046869003	Downregulated	1.25536197828995
Zfc3h1	0.238086402157184	2.99131459824916e-05	0.00371941279291096	Upregulated	1.17942722534871
Kmt2c	0.279779399777033	3.29984593141792e-05	0.00396573816582196	Upregulated	1.21400923796438
Mbnl2	0.208156005731791	3.31390490615579e-05	0.00396573816582196	Upregulated	1.15521069643122
Parp11	0.228463490191457	3.5802047142916e-05	0.00417265076396959	Upregulated	1.17158651275393
Pou2f1	0.259406917784478	3.71129812564773e-05	0.00425149818615868	Upregulated	1.19698653035954
Kmt2a	0.289562830191516	3.91327829285292e-05	0.0044075352066477	Upregulated	1.22226984567008
Ripor1	0.226865962199417	4.28036586217499e-05	0.00478081197089428	Upregulated	1.17028990721829
Ccr5	-0.527822664976671	4.33635654496955e-05	0.00480332122084519	Downregulated	1.4417516415066
Crppa	0.860438997633602	4.47406436855482e-05	0.00486430720498115	Upregulated	1.81559069262894
Cdca7l	-0.188291119974791	5.22737972171917e-05	0.00547363831329704	Downregulated	1.13941327507957
Cd74	-2.11813262236064	5.37191924586189e-05	0.00553844874248361	Downregulated	4.34131655258969
Dnah8	0.226739698619049	5.9410905410182e-05	0.00586130082494593	Upregulated	1.17018748881134
Ighm	-0.224030683213296	6.16817711024081e-05	0.00594763149701853	Downregulated	1.16799223518772
Crmp1	-0.289922410866944	6.38973541080999e-05	0.00611725883650617	Downregulated	1.22257452502388
Ccl22	-2.07445197379892	6.52725507746076e-05	0.00616090139459202	Downregulated	4.21184391701375
Kmt2d	0.287580788874346	6.5178068006672e-05	0.00616090139459202	Upregulated	1.22059178766385
Hscb	-0.217656182674353	7.27187034692023e-05	0.00670706655188551	Downregulated	1.16284288444731
Atrn	0.189811242072531	8.48736057005649e-05	0.0072456110649979	Upregulated	1.1406144715009
Icosl	-0.355074407019483	8.42742266994511e-05	0.0072456110649979	Downregulated	1.27905154668002
Dennd11	0.181004520463766	8.62697817684458e-05	0.00731818914583847	Upregulated	1.13367296496332
Gm43654	0.759562648321668	8.96512174046572e-05	0.00750997041796637	Upregulated	1.69297732240302
Birc6	0.237250742199715	9.64308158788961e-05	0.00797816188410398	Upregulated	1.17874425723352
Fasl	-0.358615460090558	0.000106306715030319	0.00858330663585163	Downregulated	1.28219479747887
Sacs	0.246814296864559	0.000119605244393602	0.00916039480347112	Upregulated	1.18658405119594
Fbxo17	0.213191833593844	0.000128863544707892	0.00964234667894321	Upregulated	1.15925008588251
Smg1	0.23123045052102	0.000128091387664237	0.00964234667894321	Upregulated	1.17383566727203
Ust	-0.230199120473766	0.00013350285798419	0.00973015087989386	Downregulated	1.17299683484257
Dnah17	0.357022579129984	0.000140260189111848	0.0101070285734736	Upregulated	1.28077990622413
Fam92a	-0.264530803389366	0.000151878290650717	0.010632566229582	Downregulated	1.20124531421906
Camk2b	-0.94241098284557	0.000156105598908193	0.0107849656812707	Downregulated	1.92173709826136
Lyst	0.191822079113364	0.000163367116859858	0.0111714768738402	Upregulated	1.14220537527204
Gm8251	0.549401683071414	0.000170624591745654	0.011434407015835	Upregulated	1.46347863373801
Dcp2	0.180480244308584	0.000175158350661203	0.0115852122637828	Upregulated	1.13326106244436
Lrp1	0.744272104659566	0.000178643513402076	0.0117370539712158	Upregulated	1.67512888890091
Nbeal1	0.239866903140941	0.00019223090504848	0.0121531642470036	Upregulated	1.18088371313561
1700007L15Rik	-0.459959047227409	0.00018782424849432	0.0121531642470036	Downregulated	1.37550277215279
Brwd1	0.182858125723117	0.000190041295518051	0.0121531642470036	Upregulated	1.13513046820497
Ogt	0.183485116351843	0.000207726845651897	0.0125980222274768	Upregulated	1.13562389947281
Nav2	0.490099285616169	0.000206121152547144	0.0125980222274768	Upregulated	1.4045415323211
Gm11707	-0.268705629005922	0.000206960004319451	0.0125980222274768	Downregulated	1.20472647460387
C920009B18Rik	0.264511640229505	0.000210826977692933	0.0127284413604432	Upregulated	1.20122935831539

Ago2	0.205233352734575	0.000214172782388808	0.0128149901890946	Upregulated	1.15287280634738
Nkg7	-0.445326246749299	0.000215308575835418	0.0128256926307649	Downregulated	1.36162199481953
Uap1l1	-0.199632385095494	0.000218249837773438	0.0128262988725233	Downregulated	1.14840569103964
Alg13	0.243056359273269	0.000219706827731398	0.0128262988725233	Upregulated	1.18349725449092
Gm11675	-0.182407770342674	0.000222093645140271	0.0128262988725233	Downregulated	1.13477617827049
Cd44	-0.189372999712374	0.000231384327236538	0.0130304375544173	Downregulated	1.14026804370408
Dse	-0.215371097308659	0.000242802738994298	0.0135032577209153	Downregulated	1.16100251491198
Cnn3	0.257939553417486	0.00025982475182191	0.0142722588060207	Upregulated	1.19576969491557
Ino80d	0.199764441607243	0.000261791009708423	0.014321571033151	Upregulated	1.14851081470498
Cpsf6	0.182554471543346	0.000265346681732558	0.0144570795742336	Upregulated	1.13489157444734
Zc3hav1l	0.279948080725355	0.000267701002521366	0.0145263017683962	Upregulated	1.21415118910147
Gm8189	0.427591940280946	0.000270298288571826	0.0146080966198717	Upregulated	1.34498673134736
Rps4l	-0.658184059121267	0.000287555661753596	0.0152872202267235	Downregulated	1.57809500231901
Bcl2l2	0.371624405075984	0.000289443242433887	0.0152872202267235	Upregulated	1.29380877714043
Vps13c	0.191316074948219	0.000296887230914758	0.0155436701404316	Upregulated	1.14180483370422
Thoc2l	0.198747690485837	0.000325915949026203	0.0163896710535543	Upregulated	1.14770167747791
2510009E07Rik	0.265118051773706	0.000327969377605319	0.0163896710535543	Upregulated	1.20173438012822
Vmn2r96	0.320512995954334	0.00032680548025174	0.0163896710535543	Upregulated	1.24877451132736
Coq4	-0.378221456618855	0.000337371768127286	0.0165742309467019	Downregulated	1.29973855965208
Srcap	0.233688217767791	0.000337593452842619	0.0165742309467019	Upregulated	1.17583711152601
Emilin1	0.316358186984768	0.000348395214286867	0.0170421206463025	Upregulated	1.24518334652046
Mss51	0.36039223475463	0.000375085929127928	0.0178906644416428	Upregulated	1.2837748782316
Armcx1	0.557433116721695	0.000379994671337778	0.0179967087630397	Upregulated	1.47164848848527
Rc3h2	0.189006654580884	0.000400592699088471	0.0183247233647876	Upregulated	1.13997853095084
Rpl34-ps1	-0.324419557893723	0.00040274049440003	0.0183603090015088	Downregulated	1.25216055324527
Gprin3	0.227712627246225	0.000445619374606277	0.0197769419796289	Upregulated	1.17097690920611
Scpep1	-0.364842542924553	0.000459710145989621	0.0202016232350783	Downregulated	1.28774107658393
Vamp1	0.256998301390874	0.000462722452625713	0.0202675458579818	Upregulated	1.19498979789154
Tbx6	0.25250218640547	0.000468111445927813	0.0203045233325905	Upregulated	1.19127144579732
Syne3	0.193208477869567	0.000498943461537608	0.021229648301551	Upregulated	1.14330353752405
Oip5os1	0.180228347406264	0.000539932767264194	0.0222729416812738	Upregulated	1.13306321049146
Ager	0.319937544019177	0.000544008167434961	0.0222976803306752	Upregulated	1.24827650836011
Tnrc6b	0.187592503390147	0.00054769632777876	0.0223804081744473	Upregulated	1.13886165447579
Atp11c	0.185387537400528	0.000557350128499258	0.0226368599159865	Upregulated	1.13712238654866
Gm13588	-0.287616183755674	0.000690379714915223	0.0264375980543107	Downregulated	1.22062173386192
Gm45223	0.545924321125794	0.000695454758122669	0.0264806253497674	Upregulated	1.4599554243506
Klrd1	-0.342870968352033	0.00070735036544204	0.0265269295431082	Downregulated	1.26827796210644
Fam199x	0.18869198884822	0.000717456922508291	0.0267113198121628	Upregulated	1.13972991772833
Zfp704	0.708102306099498	0.000732883707155431	0.0269858250741875	Upregulated	1.6336538257076
Phf11b	-0.193558817219523	0.000729010990794216	0.0269858250741875	Downregulated	1.14358120733239
Gtf2ird1	0.220724664746448	0.000797791664415332	0.0287119727259953	Upregulated	1.16531877832217
Haao	-1.01946226042196	0.000817049879197424	0.0291345514016005	Downregulated	2.02716322877839
4932438A13Rik	0.293212574785389	0.000820414989336379	0.0291671673795106	Upregulated	1.22536587088912
Irf6	0.232489964217499	0.00085457532451687	0.0300530217455303	Upregulated	1.17486090657483
Slamf7	-0.538536557229402	0.000880322678965195	0.0305672664926697	Downregulated	1.45249838268295
Gm7061	0.51581240294831	0.000899874582779734	0.0306735421915854	Upregulated	1.42979905411735
Ttll5	-0.189035196028871	0.000934508103775937	0.0317658251785062	Downregulated	1.14000108385278
Zfp871	0.196498661683244	0.000934123967797772	0.0317658251785062	Upregulated	1.14591391002619
Gm50232	0.52548455825823	0.000951874971018022	0.0320552267250114	Upregulated	1.43941695607773
Scai	0.191405624974837	0.000971746592605493	0.0323988049270931	Upregulated	1.14187570926958
Spry1	-0.24627276235068	0.000980355167215144	0.0326047153999617	Downregulated	1.18613873488548
Gm33707	0.92997343197423	0.000989359718556159	0.0327416995254523	Upregulated	1.90524090970147
Slc5a3	0.24069053617486	0.000991826129268272	0.0327424768733563	Upregulated	1.18155807084323
Sdf2l1	-0.263623580542902	0.00104329491144015	0.033857824934703	Downregulated	1.20049016182668
Gm49980	-0.867757668539857	0.00114401368159681	0.0359934633202864	Downregulated	1.82482443308528
Dpp7	-0.193770584468495	0.00120352447168332	0.0373399039212305	Downregulated	1.14374908121707
Tagap	-0.227272560191211	0.00121962368148495	0.0377520004686902	Downregulated	1.17061977913999
Cobll1	-0.341980191390502	0.00123435305149506	0.0378830583955913	Downregulated	1.26749511884976
Atf6	-0.189055584315522	0.00125032819713054	0.0381285771046687	Downregulated	1.14001719455703
Ly6c2	-0.426325227228737	0.00125647766180547	0.038187233789521	Downregulated	1.34380632629104
B130055M24Rik	0.289278925780505	0.00132655186908066	0.0394725019550343	Upregulated	1.2220293418563
Tmem263	0.205762004378255	0.00133392577304732	0.0394725019550343	Upregulated	1.15329533485488
Ptov1	0.216303228901648	0.00135304591657455	0.0397694614470365	Upregulated	1.16175288611151
Insl3	0.239442614263626	0.00140804333078116	0.0410260973096955	Upregulated	1.18053647262951
Gm7665	-0.231587808749889	0.0014074393905726	0.0410260973096955	Downregulated	1.17412646455101
Fgl2	-0.326139566121986	0.00149328194523651	0.0422245947510653	Downregulated	1.2536542929204
Lzts1	0.193452420446616	0.00154591903340102	0.0430768249577419	Upregulated	1.14349687290269
Chdh	0.254723493714652	0.00163557447633421	0.0450135620252719	Upregulated	1.19310705075394
Nipal1	0.234694751742269	0.00164472714283112	0.0451727006052573	Upregulated	1.17665775131511
Banp	0.202679925304647	0.00165753357527214	0.0453590978329723	Upregulated	1.15083414023789
Rpp40	-0.20944762415017	0.00166792495203526	0.045437394577497	Downregulated	1.15624539849279
Gm37303	0.311471503671243	0.00171257600460689	0.0460916790958759	Upregulated	1.24097280796759
Ifng	-0.330047345216184	0.00173485683821757	0.0465045724052603	Downregulated	1.25705462686194
Padi4	-0.594623111640536	0.0017833329122064	0.0472371759334038	Downregulated	1.51007804259561
Gm16120	0.534763345713087	0.00180320657515075	0.0472857946012393	Upregulated	1.44870449500778
Isoc2b	-0.208976492214041	0.0018475945500216	0.0478002718811809	Downregulated	1.15586787227926
Nf1	0.186867484639428	0.00194555360042538	0.0489235551716723	Upregulated	1.13828946937032
Mcoln2	-0.399525197235263	0.00197196558006833	0.0494023451769269	Downregulated	1.3190737213541
Gm6162	0.354874790488783	0.00198619199499699	0.0495172728320778	Upregulated	1.27887458470093
